# Habitat partitioning in Antarctic krill: Spawning hotspots and nursery areas

**DOI:** 10.1371/journal.pone.0219325

**Published:** 2019-07-24

**Authors:** Frances A. Perry, Angus Atkinson, Sévrine F. Sailley, Geraint A. Tarling, Simeon L. Hill, Cathy H. Lucas, Daniel J. Mayor

**Affiliations:** 1 Plymouth Marine Laboratory, Prospect Place, The Hoe, Plymouth, Devon, United Kingdom; 2 British Antarctic Survey, High Cross, Cambridge, United Kingdom; 3 National Oceanography Centre Southampton, University of Southampton, Southampton, United Kingdom; 4 National Oceanography Centre Southampton, European Way, Southampton, United Kingdom; Evergreen State College, UNITED STATES

## Abstract

Antarctic krill, *Euphausia superba*, have a circumpolar distribution but are concentrated within the south-west Atlantic sector, where they support a unique food web and a commercial fishery. Within this sector, our first goal was to produce quantitative distribution maps of all six ontogenetic life stages of krill (eggs, nauplii plus metanauplii, calyptopes, furcilia, juveniles, and adults), based on a compilation of all available post 1970s data. Using these maps, we then examined firstly whether “hotspots” of egg production and early stage nursery occurred, and secondly whether the available habitat was partitioned between the successive life stages during the austral summer and autumn, when krill densities can be high. To address these questions, we compiled larval krill density records and extracted data spanning 41 years (1976–2016) from the existing KRILLBASE-abundance and KRILLBASE-length-frequency databases. Although adult males and females of spawning age were widely distributed, the distribution of eggs, nauplii and metanauplii indicates that spawning is most intense over the shelf and shelf slope. This contrasts with the distributions of calyptope and furcilia larvae, which were concentrated further offshore, mainly in the Southern Scotia Sea. Juveniles, however, were strongly concentrated over shelves along the Scotia Arc. Simple environmental analyses based on water depth and mean water temperature suggest that krill associate with different habitats over the course of their life cycle. From the early to late part of the austral season, juvenile distribution moves from ocean to shelf, opposite in direction to that for adults. Such habitat partitioning may reduce intraspecific competition for food, which has been suggested to occur when densities are exceptionally high during years of strong recruitment. It also prevents any potential cannibalism by adults on younger stages. Understanding the location of krill spawning and juvenile development in relation to potentially overlapping fishing activities is needed to protect the health of the south-west Atlantic sector ecosystem.

## Introduction

Antarctic krill (*Euphausia superba*), hereafter “krill”, provide a key link between primary production and a suite of predator species [1–4]. Krill are an important grazer species in the Southern Ocean [5] with an estimated biomass of between 300 and 500 million tonnes [6]. Their importance in the diets of vertebrate predators is well documented [7] with populations of penguins, whales, seals, and albatrosses all exhibiting a dependence on krill [8–11]. Krill also play an important role in iron cycling [12,13] and carbon export [14] and support a commercial fishery, managed by the Commission for the Conservation of Antarctic Marine Living Resources (CCAMLR) [3,15].

Of the total area in the Southern Ocean (32 million km^2^), the total suitable krill habitat is considered to be approximately 18 million km^2^ [15]. The first circumpolar distribution maps of krill were compiled from data collected during the *Discovery Investigations* during the 1920s and 1930s which have been been fundamental to our understanding of krill [16,17]. These maps revealed that the circumpolar distribution is assymetric, with higher concentrations in the south-west Atlantic, the sector where the current fishery now operates. More recent studies have added further detail, showing that the sector 0^o^-90^o^W contains 70% of the total stock [18].

While the heterogeneous distribution of adult krill in the Southern Ocean is now well documented, differences in the relative distribution of the life stages within the south-west Atlantic sector are not so well known. Krill have a complex ontogeny and a lifespan of up to seven years [19]. The larval stage lasts for the first twelve months and is comprised sequentially of the following: the descent ascent development cycle which begins with eggs sinking up to 1250-1850m through the water column [20] until they hatch as nauplii and begin swimming back to surface waters; the metanauplii stage (lasting nine days at 0.5 ^o^C [21]) where the larvae remain in a non-feeding state; the calyptope stages when the larvae feed for the first time; and the furcilia stages which outlast the first winter. After the furcilia stages the krill moult to post-larval juveniles, continuing to grow to adulthood in subsequent years. A better knowledge of the relative distribution of these different life stages would help us to understand the regional distribution of spawning and recruitment [17], identifying hotspots that may be sensitive to influences such as climate change and fishing.

The *Discovery Investigation* [16,17] krill density maps typically presented the data as a series of overlain bubble plots of abundance, based on a composite of surveys spanning over a decade. Although these composite maps are hard to draw quantitative insights from, they are still valuable, and continue to be used as a source reference for the distribution of each life stage [15]. However, since then the environmental system has changed, with warming and a changing suite of krill predators, and furthermore a wealth of data has been collected in the modern era since the 1970s. These include a series of large-scale, semi-synoptic surveys that have been used to determine the distributions of krill, including FIBEX 1981 [22], SIBEX 1984–85 [23], CCAMLR 2000 [24], Southern Ocean GLOBEC 2001–2005 [25], US AMLR 2011, and Palmer LTER [26]. In addition, a series of maps of krill life stage distributions (some including larval stages) have been produced from single surveys [27–32]. All of these studies are valuable to our understanding of krill but, being based on one or a few surveys, they are spatially and temporally limited and provide only snapshots of krill life stage distribution, each of which can vary substantially between surveys [33,34]. There has been no attempt, so far, to map the relative distribution of each krill life stage using all available modern era data, equivalent to the old *Discovery* approach.

The overall aim of this paper is to quantify and compare the distributions of all the life stages of krill within the south-west Atlantic sector during the modern (post 1970s) era. The approach is similar to that of the “*Discovery Investigations*” in combining all available data for each life stage from multiple surveys to produce a “climatology” map of mean distribution. The much greater volume of data available since the 1970s allowed us to plot the data quantitatively as mean densities within grid cells. To achieve this we compiled all available egg and larval krill abundance data from the modern era into a single new database. For the postlarvae we integrated the data provided from two existing databases, namely KRILLBASE-abundance and KRILLBASE-length frequency. Our first objective was to identify krill spawning hotspots to highlight potential source regions for krill recruitment. The second objective was to compare the relative distributions of the life stages to investigate whether the habitat was partitioned during the summer season. Distributions of both krill and krill sampling are heterogeneous so, in addition to the gridded maps, we included simple “habitat analyses” based on water depth and mean temperature to provide alternative visualisations of the distribution of each life stage.

## Methods

### Overview of the krill databases used for this study

In this analysis we combined two existing post-larval krill databases, entitled KRILLBASE-abundance and KRILLBASE-length-frequency (KRILLBASE-lf) and compiled an additional database on density of the eggs and larval stages specifically for this study. The source data compiled for this new larval dataset is detailed in S1 Table. Each of the three databases is a large, multi-national composite of net sampling data, summarised in Table 1.

**Table 1:** Summary of the three composite databases that were used for this study.

Attribute for each database	KRILLBASE-abundance	KRILLBASE-length-frequency	KRILLBASE-larvae
Source data	DOI: 0.5285/8b00a915- 94e3-4a04-aa903-dd4956346439	Held at the UK Polar Data Centre at the British Antarctic Survey, Cambridge	Compiled for this study
Reference on data source for further information	[35]	[6]	(see S1 Table)
Summary of database	All available un-targeted scientific net catches. Measured in density (no. m^-2^)	Length, sex and maturity stage data of post-larval krill	Densities of eggs, nauplii, metanauplii, calyptopes and furcilia larvae
Years of coverage after screening	39	40	21

All three of the databases have been compiled in a similar manner, with information gathered from a variety of sources ranging from paper logbooks to published reports and institute records that were sent to us. Since the 1920s and 1930s of the main *Discovery* era there is a long gap in data available to us and the first record of what we define in this paper as the “modern era” was in 1976. This modern era of data comprises 41 years spanning 1976–2016.

**Fig 1:**
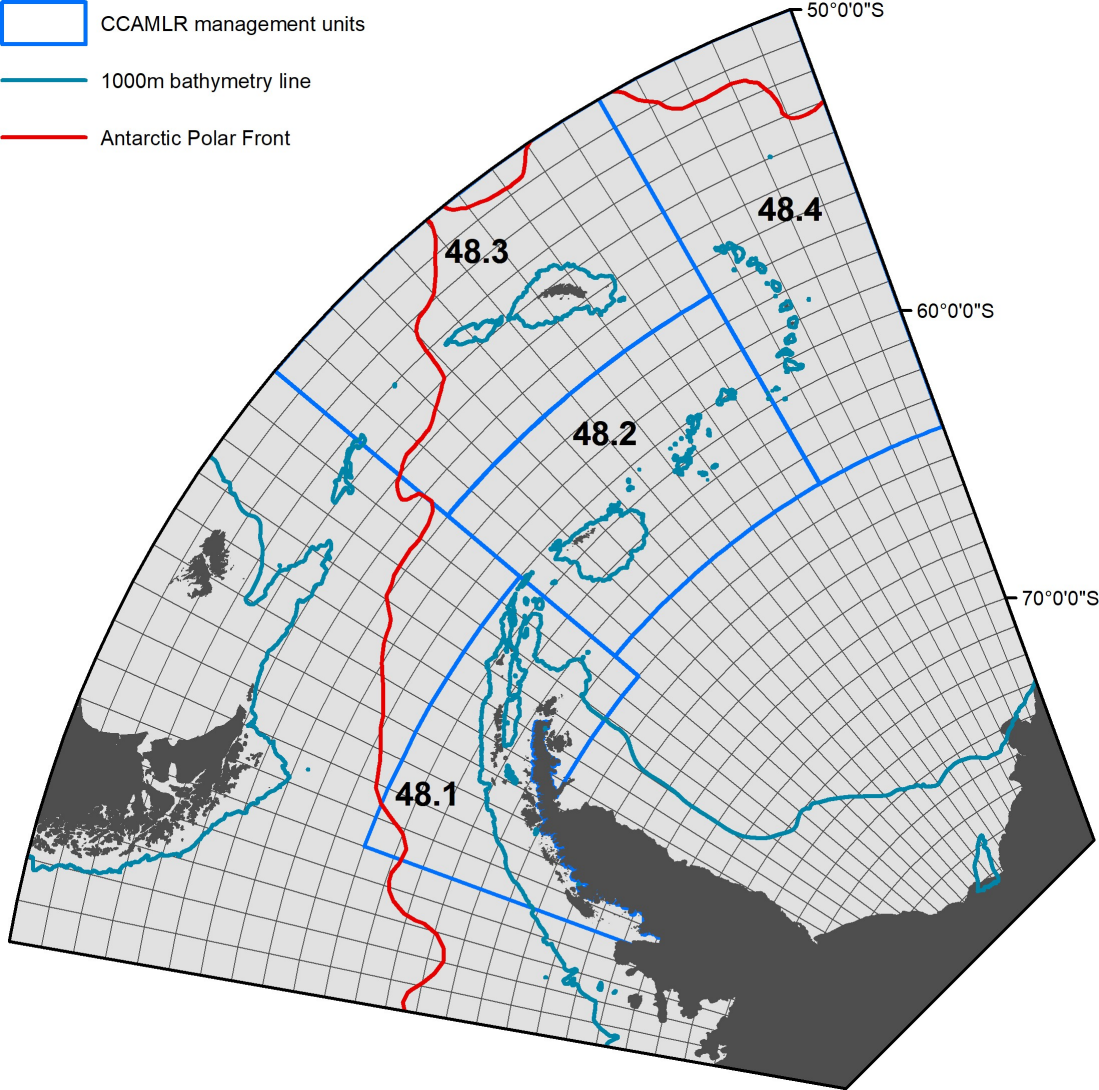
The study area. Also depicted are CCAMLR management subareas (each of which has its own catch limit), 1000m bathymetry line, Antarctic Polar Front and the 1˚latitude by 2˚ longitude grid cells used for this study.

The specifics of screening of the individual databases are described in the following sections. For all three databases the data were pre-screened to include only the south-west Atlantic sector of the Southern Ocean, defined as between 20° and 80° W. The northern limit of data extracted from the three databases was determined by the position of the Antarctic Polar Front. Its position is based primarily on ref [36]–see Fig 1. The Southern limit was determined by the coast of Antarctica. All data were plotted using ArcGIS version 10.2.2. A few data points in the length frequency database plotted on land and were removed. Table 2 summarises the data coverage provided by the three databases after all of the screening procedures. S2 Table shows the data coverage of the three databases by year. S1 Fig and S1 Table, S3 Table, S4 Table and S5 Table contain the source data used to create all the figures in the main text.

**Table 2:** The number of stations, per approximate 10-year period, provided by each of the data sets after screening. The lower numbers of stations in the decade 2006–2016 reflects an increase in the use of acoustics and a decrease in funding for large scale surveys. S2 Table provides further breakdown of stations into early and late austral season coverage for every sampling year.

Number of stations	1976–1985	1986–1995	1996–2005	2006–2016	TOTALS (after screening)
KRILLBASE-abundance	1682	1522	3153	1270	7627
KRILLBASE-length-frequency	1745	1040	1425	184	4394
KRILLBASE-larvae	776	11	524	312	1623

#### Allocation of data into early and late season

The large majority of the KRILLBASE datasets were from the austral spring-summer-autumn season (i.e. from November to March). Only a few records were in October or April, and only the larval database had any records in May (29 stations).

We aimed to use a wide span of coverage (October to May) as we wanted to maximise the available sample size to provide a robust comparison between the early and late parts of the season. The sample numbers were indeed small from each ends of the season so in reality they had little effect on our grid-scale averaging. Giving the fact that there was no clear reason to exclude them we therefore chose to include them.

For this paper we defined “year” as austral season, such that, for example the year 1980 spanned October 1979 to May 1980. Within each year we further divided all data as being either “early season” (defined here as 1 October–December 31) or “late season” (1 Jan– 31 May). The majority of the larvae were recorded in the late season, thus providing a natural division for our analysis of habitat partitioning, while retaining large sample sizes.

#### KRILLBASE-abundance database

KRILLBASE-abundance (https://www.bas.ac.uk/project/krillbase) is an open access database of net-based juvenile and adult (post-larval) Antarctic krill and salp densities [35]. Because the database is a composite of multiple sampling methods with variable efficiency in catching krill, all densities (nos. m^-2^) used in this analysis have been standardised to account for variation in sampling method. This procedure is detailed in Appendix 1 of [18] and its rationale is further explained in [35]. In brief, we used the KRILLBASE abundance dataset to derive a series of conversion factors based on the net mouth area, sampling depth, time of day and time of year of sampling. These factors were used in an empirical model to multiply the catch values for the non-zero krill densities to those of a single and relatively efficient net sampling method; namely a night-time RMT8 net sampling from 0–200 m on 1 January. Prior analyses [35] have showed that this standardisation procedure yields a very similar geographic pattern of krill distribution to that based on un-standardised krill densities, although mean values were overall higher.

For our study, we filtered the database for standardised numerical densities of post-larval krill that fell within the previously outlined spatiotemporal parameters, with additional filtering on sampling depth following Atkinson et al., (2008). Namely, the upper sampling depth was within the topmost 20m and the bottom sampling depth was at least 50 m depth. This resulted in a total of 7,627 usable stations out of a possible 12,758.

#### KRILLBASE-length-frequency database

The full KRILLBASE-length frequency database contains the individual length measurements for >1,000,000 krill, with sex and maturity stage as additional variables for a portion of these. These values were obtained from both scientific hauls and commercial fisheries. Most of these were oblique or targeted hauls (often towed horizontally) within the top 200m layer. Unlike the other two databases we included horizontal hauls in this study to enhance sample sizes, based on our unpublished prior analyses that showed that the trends in krill length were congruent between the two sampling methods. After filtering for the spatiotemporal criteria stated in section 2.1, we ensured that any records outside of the following parameters were removed: Krill <15mm were excluded, since krill this size would likely be furcilia, counted in the larval database. Records sampled with a >6mm mesh were excluded due to the possibility of net mesh selection and under-representation of the smaller krill. This removed both commercial trawl data and some of the scientific trawls. Data from some scientific nets were further excluded where mesh size was not stipulated, including nets described as trawls. This left 530,018 measured krill from 4,394 stations.

#### Egg and larval database

We collated the larval database for this study and it contained density estimates for eggs and individual larval stages. The component surveys and sampling details are described in S1 Table. These data were not standardised in the same way as the postlarval density data, given the decreased net escape responses of the small larvae. Further, the nets compiled were restricted to those that provided a reasonable estimation of egg and larval densities (i.e. 100–500 μm. mesh mesozooplankton nets–see S1 Table). An important difference in the screening compared to the postlarval data was the requirement for the top depth to be the surface and the bottom depth to be at least 200 m, to ensure that the densities of calyptopes and furcilia, known to undergo extensive diel vertical migrations, were represented consistently. We stress that sampling even in the whole of the top 200m layer will under-sample eggs and nauplii due to the deep developmental cycle, and discuss this issue in section 4.1.

Our compiled larval database contains detailed information on densities of a range of early life stages, and includes depth distributions of life stages from stratified hauls. We combined the results from stratified hauls at each station to provide densities (no. m^2^) within the whole sampled water column, and for four larval stages; eggs, nauplii plus metanauplii (hereafter referred to as “nauplii”), calyptopes, and furcilia. Low data availability and the fact that larvae were relatively rare before January meant that we could only provide late season distributions for these four larval stages. For our analysis we extracted 1,623 usable stations out of the total 3,449 in this database.

### Producing grid maps of krill life stage densities

Baseline maps of land masses were plotted using the WGS 1984 Antarctic Polar Stereographic co-ordinate system using Arc GIS v 10.2.2. To these maps, a grid of cells, 1^o^ of latitude by 2^o^ of longitude, and the Antarctic Polar Front position was overlaid (Fig 1). By joining each of the three databases separately to this grid, we derived a series of mean krill indices for each grid cell, both for early and late season where data allowed. These indices included densities for each of our four larval groups (eggs, nauplii plus metanauplii combined, calyptopes and furcilia), as well as total post-larval density. From the KRILLBASE length frequency database extracted for each grid cell, we determined the fractions of juvenile and adult krill (defined respectively as 15–30 mm and > 30 mm following ref [26]) and the sex ratio for these adult krill. By multiplying the appropriate indices for each cell we calculated the mean density of female and male adult krill, juvenile krill, in addition to those of the four larval groups. Calculations of juvenile and adult densities could only be made if there were values from both the density and length-frequency databases in any given grid cell. This led to 389 grid cells being excluded due to lack of length frequency data, and 41 grid cells being excluded due to lack of post-larval density data. These exclusions obviated the need to interpolate between grid cells.

### Selection of environmental data

One of our objectives is to use environmental descriptor data, alongside the plots themselves, to illustrate the degree of habitat partitioning among krill life stages. We stress that krill life stage distribution has been linked to a wide range of environmental factors such as sea ice, phytoplankton concentration, oceanographic fronts, eddies, bathymetry and temperature [4]. It is beyond the scope of this paper to examine how all of these the factors influence the distribution of each life stage. In any case, the timespan of the data pre-date the satellite era, and matching environmental data, specifically chlorophyll data, are unavailable for most of the 41 sampling years. Instead, for this analysis we selected both mean water depth and mean water temperature within each grid cell as our habitat descriptor variables. While water depth is clearly invariant across the 41-year timespan of observations, mean water temperature changes both seasonally and over longer timescales. The purpose of this temperature index was therefore to provide an internally consistent index to portray the radically different distribution patterns of the life stages across the sector.

To derive these two habitat descriptors, ocean bathymetry was sourced from the GEBCO data series. The most up-to-date GEBCO data available was version GEBCO_2014 grid (The GEBCO_Grid, www.gebco.net). These data were used to create isobaths and to derive mean water depth for each of the grid cells used for analysis in ArcGIS. Water depth can be used as a rough proxy for a grid cell’s position relative to land; 0-1000m being the shelf region, 1000-2000m the shelf break/slope, 2000-3000m off shelf, 3000-4000m being oceanic and >4000m ocean trench. To derive mean sea surface temperature (SST) for each grid cell, data from 1 January 1979–1 December 2014 were downloaded from the European centre for medium-range weather forecasts, specifically the European re-analysis interim dataset. An average February value was taken for our study area, as described in [35], it contains the least ice cover.

### Environmental habitat analysis

As described in section 2.3 there are numerous variables which may influence the distribution of Antarctic krill [18,37–39]. We sought to summarize key physical habitat characteristics with a simple index which combines water depth and climatological mean temperature since these portray the differing habitats of each krill life stage. For our water depth and temperature analyses, we first obtained mean water depths and temperatures for each cell of our 1^o^ latitude by 2^o^ longitude grid. For each sampling station we then related each available life stage density to its grid mean temperature and depth value. This matrix of linked krill and physical data was then divided into broad categories of temperature (-2-0 ^o^C, 0–2°C, 2–4°C, > 4°C) and of depth (0-1000m, 1000-2000m, 2000-3000m, 3000-4000m, >4000m). While other finer divisions were trialled, these broad categories preserved relatively large sample sizes, while still being able to depict the large differences in habitat that we found between the life stages. Within each of these combinations of temperature and depth we calculated the arithmetic mean krill density for each of the life stages.

## Results

### Overview of sampling coverage and life stage distribution

**Fig 2:**
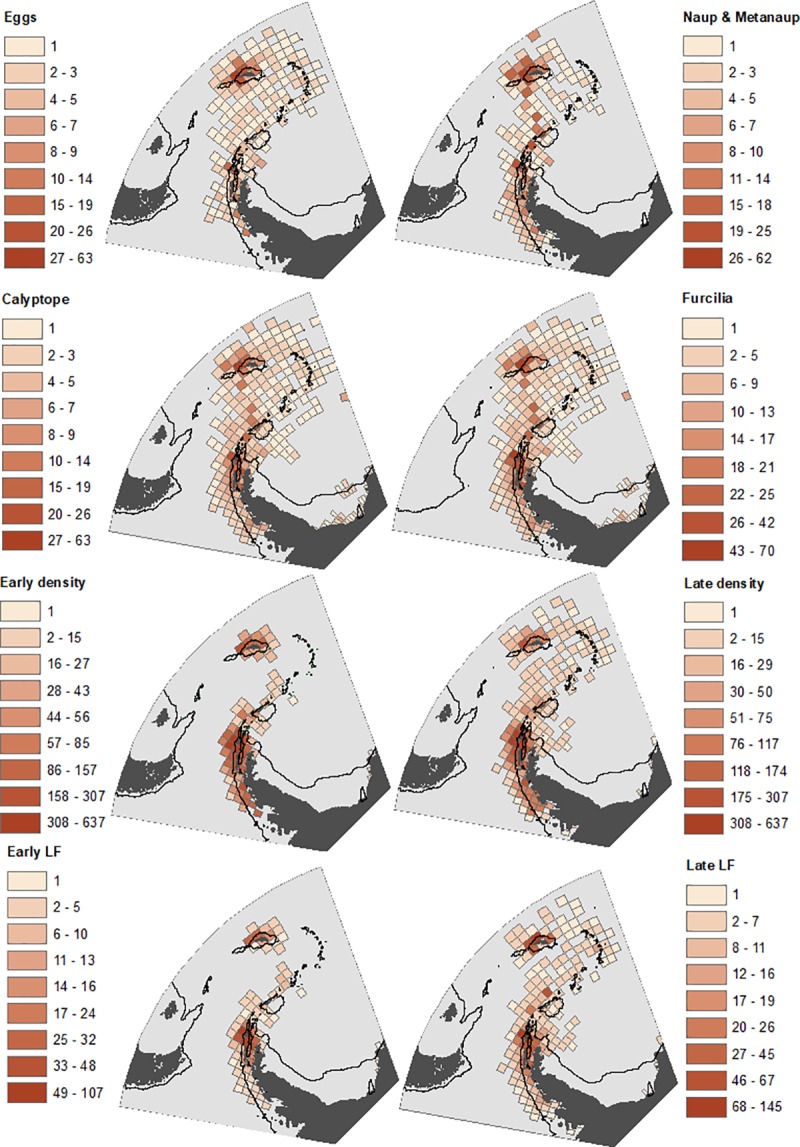
Density of sampling coverage for each krill life stage. Natural breaks have been used for the scale division of number of stations per grid cell: note the difference in scale between life stages. Eggs, nauplii, calyptopes, and furcilia are depicted here and in the rest of this paper only for the late (January to May inclusive) part of the survey season due to lack of stations and low abundances in the early part of the season. Sampling intensity plots for combined juveniles and adults male and female density and length-frequency (LF) are shown both for the “early” (October to December inclusive) and “late” (January to May inclusive) parts of the survey season. Both the 1000m isobath (continental shelf edge; solid black line) and the Antarctic Polar Front (dashed black line) are shown.

**Fig 3:**
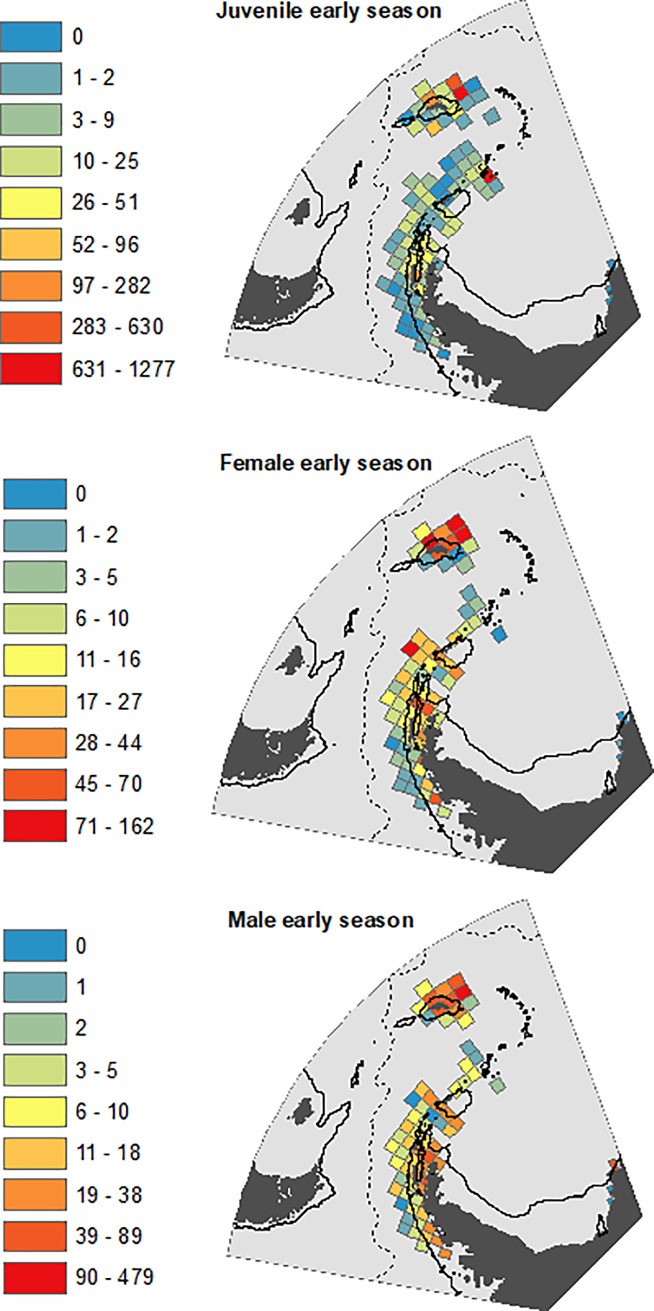
Distribution of krill life stages in “early” season (October-December). Scale bars of mean density (no. m^-2^) in each grid cell differ between the life stages. Natural breaks are used for scale divisions. Grey represents un-sampled areas. Both the 1000m isobaths and Antarctic Polar Front are as Fig 1.

**Fig 4:**
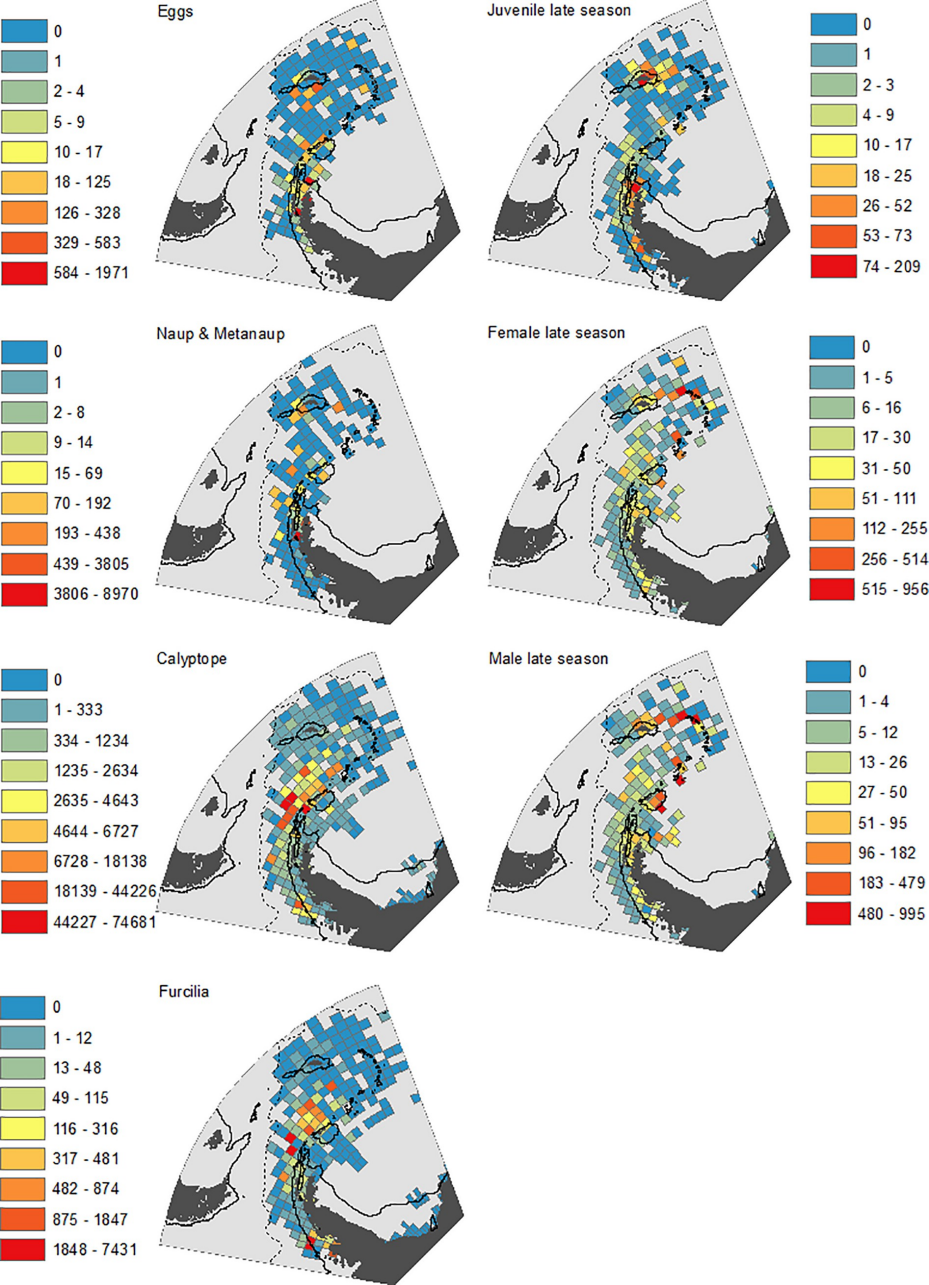
Distribution of krill life stages in “late” season (January to May). The map layout is identical to that in Fig 3.

Temporally, the most intensively sampled period in our analysis was 1996–2005 (5,102 stations), with the least sampled being 2006–2016 (1,766 stations) (Table 2). Spatially, sampling was widespread across the south-west Atlantic, albeit with most emphasis on shelf and oceanic waters surrounding the Scotia Arc, particularly for the length frequency data (Fig 2). We have compared the relative distributions of the life stages both during the early season (Oct-Dec) (Fig 3) and the late season (Jan-May) when larvae were abundant (Fig 4). The most striking features of the maps were first, the general northward and simultaneous horizontal spread of the larval stages, from eggs to furcilia; second the relatively restricted and off-shelf distributions of calyptopes and larvae; third, the highly restricted shelf distribution of juveniles, particularly in the late season; and fourth the much more extensive distributions of the older (adult) males and females. Thus for example, 58% of all grid cells had an average density of zero for late juveniles, while the late season males and females had respective values of only 14% and 11%.

### Distributions and habitat analysis

**Fig 5:**
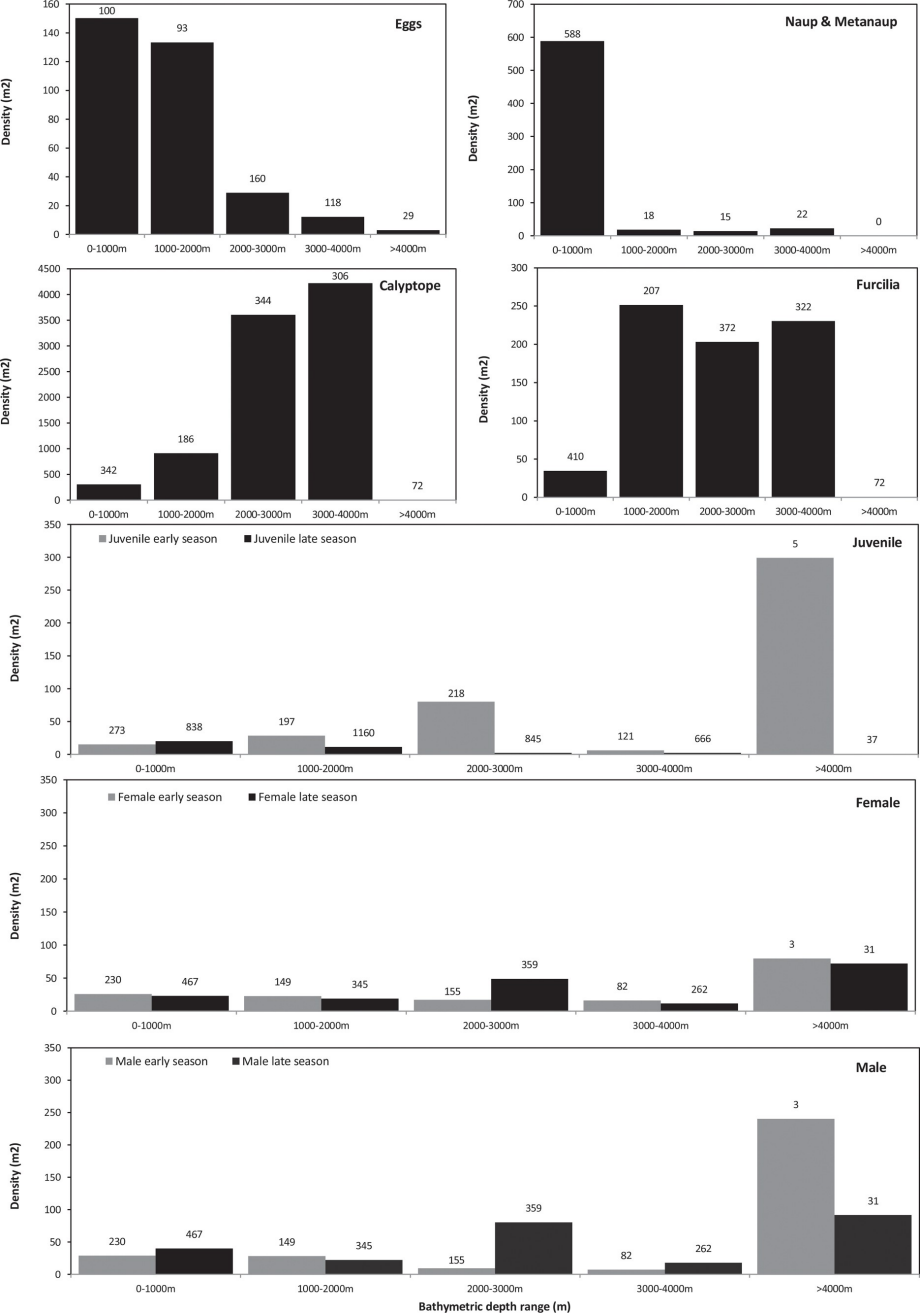
Mean densities of each life stage in relation to water depth of their sampling location. Note variation in scale of y-axis. These values were based on arithmetic mean density of all krill stations located within each water depth category. Number of stations contributing to each depth range is provided above the bars, to emphasise the low sampling density in the deepest environments–the narrow ocean trenches which lie adjacent to the Scotia Arc. Grey bars are early season (October-December), black bars are late season (January to May).

**Fig 6:**
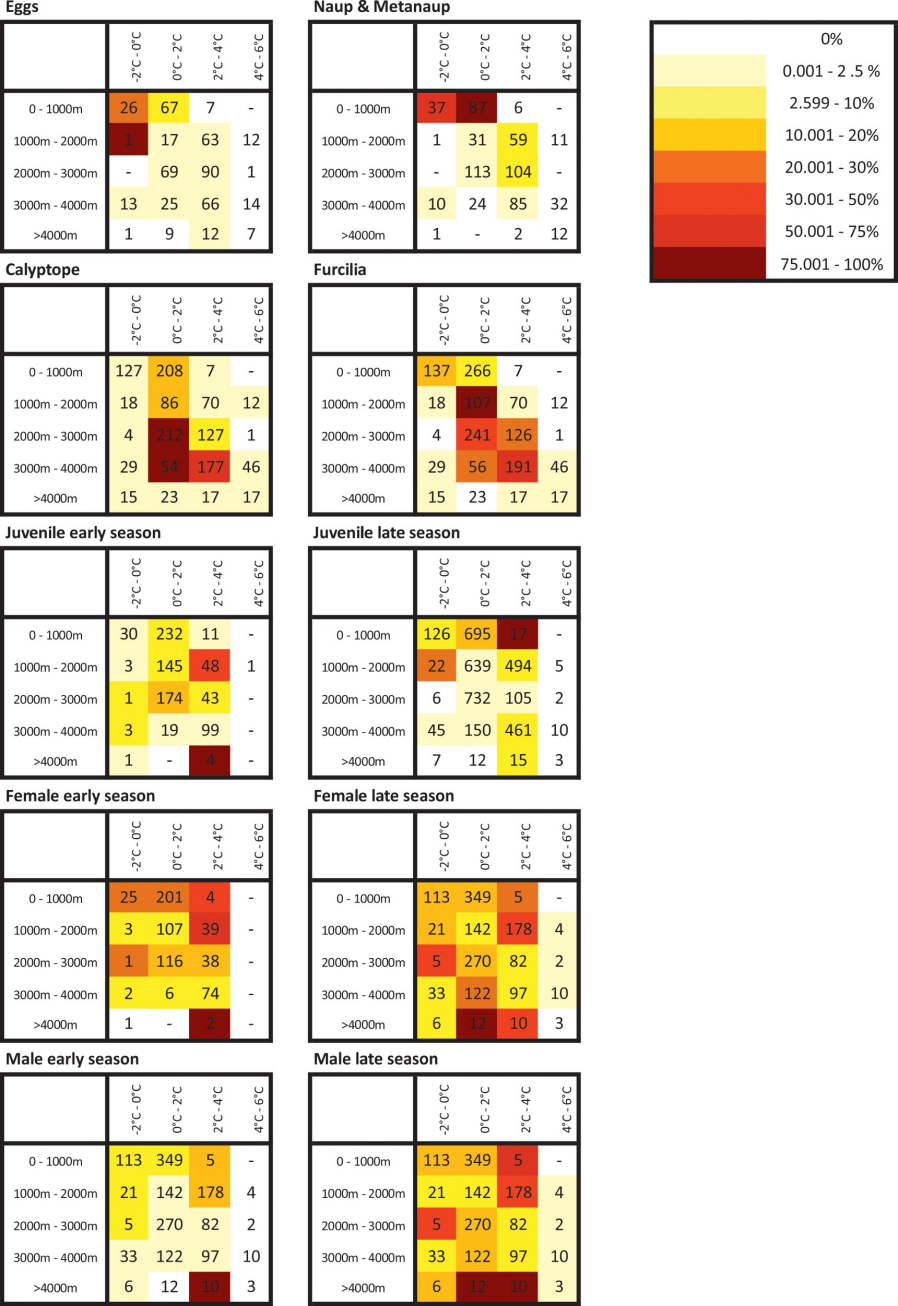
Relative densities of krill life stages in relation to water depth and temperature. To help comparison across the multiple life stages, the colour coded values within each combination of water depth and temperature are normalised by calculating them as percentages of the maximum value (i.e. the water depth-temperature combination with the highest mean krill density). “Early” denotes the period October-December inclusive. “Late” is January to May. Number of stations contributing to each density estimate is provided for each cell.

Given the fundamental differences in bathymetric distributions of the stages evident in Figs 3 and 4, we have plotted their mean bathymetric distributions in Fig 5. We then illustrated the distribution patterns in terms of depth and temperature combined (Fig 6). The patterns derived from these various depictions of distribution are described in detail below, in the sequence from spawning females, through larvae to juveniles.

#### Adults

Highest densities of adult krill, for both sexes and in both the early and late parts of the season, tend to be in the vicinity of the Scotia Arc. These elevated densities occur both over the shelf and over the deep trench environments that lie adjacent to the Scotia Arc. (Figs 3 and 4). When considering the effect of our other methods of depicting these data (Figs 5 and 6) the overall abundances and distributions of adult males and females are similar, being found across a broad range of water depth and temperature combinations. However, there is some evidence for a seasonal change with respect to bathymetric distribution, this being congruent across both sexes. In the early part of the season before January, males and females are most abundant near shelf and slope waters, whereas in the late season the highest densities of both sexes are seen off shelf. (Fig 5).

#### Eggs and nauplii

In contrast to the wide distribution of adults, the greatest densities of eggs and nauplii appear over or close to the shelves near the tip of the Antarctic Peninsula and the South Orkneys, with isolated occurrences in the Scotia Sea and south of South Georgia (Fig 4). Figs 5 and 6 show a strong association of both of these stages with cold shelf and shelf-slope waters.

#### Calyptopes and furcilia

Calyptopes and furcilia had similar distributions, but these were very different to those of all other life stages. Their highest densities were in oceanic waters of the Scotia Sea, stretching from the tip of the Antarctic Peninsula eastwards across the southern Scotia Sea. High densities were recorded during a 2011 survey in the Marguerite Bay area (S1 Table) and these are reflected in a secondary high density area in this area in the composite maps of Fig 4. Overall, both the calyptopes and the furcilia were found most commonly in habitats with deeper and warmer water than found for the eggs. This is well illustrated in both of our habitat depictions (Figs 5 and 6); highest densities in 2000-400m water depth and 0–2°C temperatures that are characteristic of the central/southern Scotia Sea.

#### Juveniles

To provide a comparison with the eggs and larvae data presented for the late season of January-May, we start by describing the late season distribution of juveniles. There are clear population centres evident in Fig 4, both at the Antarctic Peninsula shelf and around the South Georgia shelf. Exceptionally high juvenile densities seen at South Georgia have been associated with several notable spawning successes, for example in 1981 and 1996. Summaries of the habitat (Figs 5 and 6) support a strongly shelf-centred late-season distribution.

By contrast, interpreting the early season distribution of these 15-30mm juvenile krill is not as straightforward, as these krill may represent a mixture of year classes. In October-December the smallest in this size range may be nearing 1- year old (i.e. 0+ age class) whereas the largest may be one year older (1+ age class). Whatever their age, they have a clearly more oceanic distribution (Figs 5 and 6), albeit with the same broad range of habitat temperatures as late season juveniles. This movement of the juvenile distribution onto the shelf through the season is thus opposite in direction to the off-shelf movement of adult males and females.

## Discussion

Our analysis based on composite data collected over multiple surveys spanning 41 years provides overview maps similar in style to the large scale distribution maps from >80 years ago [16,17], an era when most of the large baleen whales had already been killed and when temperatures were cooler [40]. We believe that a synthesis of these krill life stage distribution data from the modern era into a series of quantitative maps is useful for a number of reasons. First it brings the wealth of larval krill data collected over the last 41 years into one place, to allow comparison with existing compilations of postlarval krill data within KRILLBASE [35]. Second, it allows the identification of specific habitats that may be particularly sensitive for krill, for spawning, nursery of larvae or for recruitment, allowing improved spatial planning for fisheries management or conservation. Third, given the ongoing rapid climate change within this region, we hope that the data and maps can be used for future efforts to model past, present and future krill distributions and how these may respond to environmental change [37,38,41].

### How well do composite maps represent krill life stage distributions?

While net sampling has contributed much to our knowledge of krill, there are several factors that might affect the quality of data collected. Avoidance [3,16,42], escapement and damage [43] and bias towards certain life stages [25] all affect net sample data. Previous studies [6,44] have argued that there is significant under sampling of the juvenile stage of krill, potentially because of avoidance [3] and the mesh size used for nets [45]. For the younger stages, the early developmental descent and re-ascent cycle [46] will mean that only a portion of the standing stocks of eggs, nauplii, metanauplii and even calyptopes will be retained by net sampling, which is typically only to 200 m depth (S1 Fig). Even the post-larval stages make extensive vertical migrations to over 3000m, leading to underestimates of density based on net sampling in the upper layers [47,48]. Targeted tows and acoustics have shown very high krill densities within bays and fjords [49,50], but due to logistical complexities these areas are generally not sampled in surveys with untargeted tows. For all of these reasons, the emphasis of this study is on horizontal patterns of abundance of each stage within the surface layers rather than comparisons of densities across life stages.

Our maps are generated from composite data collected over many seasons and should not be interpreted as single-season snapshots. They are thus akin both to the *Discovery* maps and more recent coarser-scale composite maps of the circumpolar distribution for calyptopes, furcilia and postlarval krill [4]. A number of large-scale, short-term synoptic surveys have been used to determine the distributions of krill (FIBEX 1981, SIBEX 1984–85, CCAMLR 2000, Southern Ocean GLOBEC 2001–2005, US AMLR 2011). These examples form part of the composite database analysed here (S1 Fig). Larval distributions, in particular, vary between surveys such that some of these surveys provided similar distributions to ours and others did not.

A potential issue for all distribution maps is the uneven distribution of sampling effort. To address this, all of the data have been averaged by grid cell to provide estimates of mean no. m^-2^. However, given the patchiness of krill, the precision of these mean values will inevitably be much lower in the less intensively sampled oceanic areas. This leads to juxtaposed grid cells with high and low calculated mean density in the distribution plots of Fig 3 and Fig 4. Notwithstanding these various issues of sampling, our maps and data visualisations (Figs 3–6) are based on thousands of sampling stations over multiple years (Table 2) and thereby provide the best available overview of relative distribution of krill life stages from observational data over the last 41 years.

### Are there spawning hotspots and larval nursery areas?

The calyptope and furcilia stages occur mainly off the tip of the Antarctic Peninsula in the south Scotia Sea, and with a secondary area in Marguerite Bay off the Western Antarctic Peninsula (WAP) which revealed high densities during a survey in 2011[32]. Importantly, these areas of elevated densities of calyptopes and furcilia are much more restricted than those of adult krill or eggs. The reasons why calyptopes and furcilia are located only in parts of the adult distributional range are unclear. It has been suggested [51] that the presence of warmer Antarctic Circumpolar Current (ACC) water along the Antarctic Peninsula speeds the development time of eggs, allowing the hatched nauplii to return to the surface waters with greater energy reserves, giving them more time to find food. This interpretation suggests that the chance of an egg reaching the calyptope stage is dependent on having suitable environmental conditions. Temperature has already been shown to have an impact on the developmental capacity on the early larval stages [52,53]. The metanauplii stage is the last non-feeding stage; once they have metamorphosed into calyptopes they must be able to feed. So one explanation for the relatively localised larval krill distribution is that they exhibit both suitable environmental conditions for rapid egg development [51,52], coupled to high levels of the correct food sources both for spawning females [54] and for the calyptope and furcilia stages.

The next conundrum is where the eggs that successfully reach the furcilia stage were spawned. Our density maps (Fig 4) and analyses (Figs 5 and 6) show that the greatest densities of eggs, nauplii and metanauplii are found on the shelf or shelf slope around the tip of the Antarctic Peninsula and the South Orkneys. Importantly, we found very few eggs present in the areas where there were high densities of calyptopes and furcilia. One explanation for this is a disproportionately high mortality of eggs laid over shelf habitats, either through sinking to the seafloor and being unable to undertake the developmental cycle or due to elevated predation [55].

An alternative (and non-mutually exclusive) explanation for the disconnect between the distributions of feeding and non-feeding larval stages is that eggs laid near the shelf edge were being advected offshore, in a general northerly and easterly direction [16,56]. Modelling of particles released along the continental shelf break to the west of the Antarctic Peninsula shows that they are carried north-northeast by Ekman drift into the path of the fast flowing ACC [57–59]. Modelled trajectories of larval krill across the Scotia Sea yielded maps of distribution [60] that are similar to those presented here. Importantly some models have also shown that the speed of particle advection allows enough time for larval development. It has been suggested [57] that krill from the Antarctic Peninsula could potentially reach South Georgia within 140–160 days. Therefore, we suggest that the presence of calyptopes and furcilia in the middle of the Scotia Sea is the result of spawning on the continental shelf break region off the tip of the Antarctic Peninsula and subsequent advection. As these life stages develop, they are advected further off shore by the hydrographic regime.

Calyptopes and furcilia have also been found around Marguerite Bay at the base of the WAP. The importance of Marguerite Bay for krill populations has been noted in the past [61–63]. Interestingly the hydrographic systems along the WAP mean that Marguerite Bay is a retention point for krill [64,65]. Further modelling work has shown that particles released as far north as ~65°S along the WAP will be moved southwards in the coastal current [58]. This evidence suggests that eggs spawned along the WAP (as far north as ~65°S) could end up within Marguerite Bay. It is also possible that these larvae originate from local spawning, but there are no data to corroborate this.

Notwithstanding our uncertainties over spawning locations, the evidence from the overall distributions of adults, eggs, nauplii and metanauplii suggests that spawning occurs over wide areas including both shelf and adjacent deep water habitats, and from the Antarctic Peninsula up to South Georgia. However, the much more restricted distribution of calyptopes and furcilia suggests that many of these advected early stages fail to reach the feeding larval stages.

### Partitioning of habitat by different life stages

**Fig 7:**
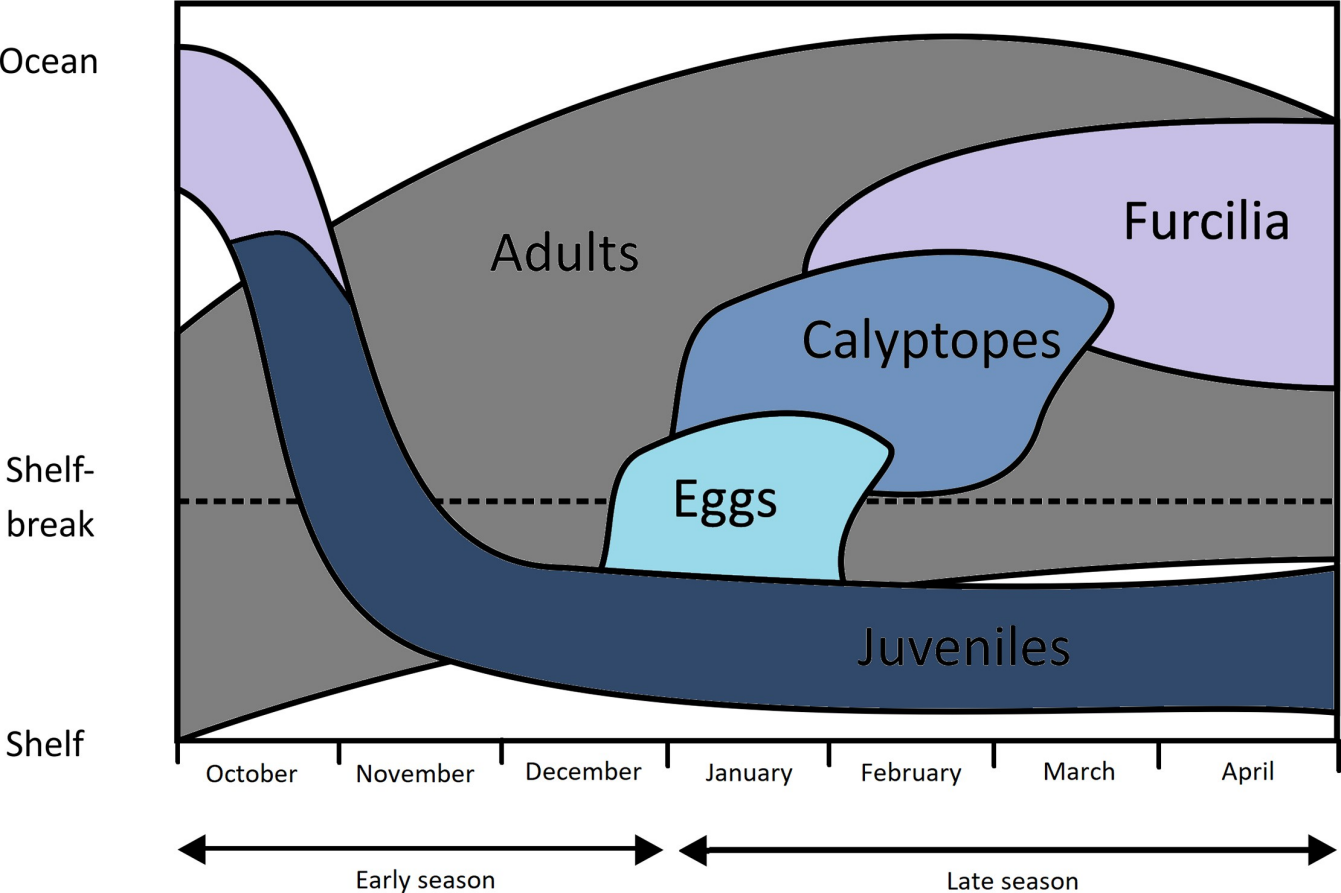
Schematic of seasonal change in onshelf—offshelf distributions of krill life stages. This illustration is based mainly on our Fig 5. The schematic portrays the main areas of the distribution relative to the shelf throughout the austral spring to autumn period. It builds on a schematic published in Fig 2.6 of [4], but includes the observed redistribution of juvenile krill from oceanic waters back to shelf waters throughout the austral spring. This schematic simply reports the changing distributions seen in our study and does not propose candidate mechanisms such as advection, migration or differential mortality.

We found strong evidence for habitat partitioning, visible both on the distribution maps themselves and through the use of water depth and mean temperature as simple habitat descriptors (Figs 5 and 6). While these are not complete descriptions of the krill habitat (which would include sea ice, food levels and other factors) they are sufficient to illustrate clearly that differing spatial distributions of the life stages exist. We have attempted to trace the strong differences in distribution between the life stages and between the early and late halves of the austral summer season in Fig 7. This illustrates the spatially-separated distributions of the larvae and juveniles, as well as the opposing on-shelf–off-shelf shifts in distributions of adults and juveniles throughout the season.

The strong on-shelf–off-shelf divide between larvae and juveniles is clear in all our depictions of distribution. Eggs were found in greatest densities in cooler, (-2°C—0°C) shallower waters (1000m-2000m) than furcilia, which were found in warmer (0°C-4°C), deeper water (2000m-4000m). This partitioning could be driven partially by the hydrological processes described above, that advect developing larvae from regions of egg production into downstream oceanic waters of the Scotia Sea. The survival of the calyptope and furcilia in the offshore region will likely also depend on food availability. This was highlighted by previous research that modelled the transport of larval stages across the Scotia Sea and found that it could only be successful under certain food conditions [60].

Food availability is thought not only to determine the success of larval stages but, through intraspecific competition for food, large scale population fluctuations in adults [66]. Across this whole sector, the density of postlarval krill can vary both coherently and greatly between years [1] reflecting high recruitment success only once or twice per decade [67,68]. This episodic recruitment leads to episodic one or two-year periods with enormous numbers of small krill (e.g. 1981/1982, 1996/1997). For schooling species, local grazing impact may be intense in years of high abundance, and only partially alleviated by dietary diversity [1] and by their nutrient excretion and fertilisation effects [13]. During years of exceptionally high abundance of larval and juvenile krill, the partitioning of the main habitat between the life stages would reduce this competition for food.

In addition to its role in reducing food completion among the numerous early life stages of krill, habitat partitioning may also have other advantages. It would reduce cannibalism, recorded in post larval krill in the laboratory (49,71, 74) in the field [69], and in larvae in the laboratory [70]. It may also have advantages in food acquisition. Some studies report a shelf-ocean gradient in phytoplankton size with microphytoplankton (20–200μm) dominating the onshore regions and nanophytoplankton (2–20μm) in offshore regions [71–73], in which case the small and larger life stages may have access to differing size ranges of food. While the feeding behaviours of the life stages (including cannibalism) have seldom been compared, differing distributions of the life stages may have advantages beyond the reduction in intraspecific competition.

Whatever the advantages, the mechanisms behind the differing distributions of furcilia and juveniles are difficult to explain based on our current understanding of krill. The furcilia show a very clearly-defined distribution over waters between 1000 and 4000m deep and 0°C-4°C; these are characteristic of the Scotia Sea. In contrast, the juveniles have a strong affinity for shelf and shelf slope habitats (0-2000m) which are both upstream and to the south. As our main distribution patterns are compared for the “late season” period (i.e. January–May), this means that these late-season juveniles will be one year older than the corresponding calyptopes and furcilia. This suggests that, during this intervening year, the developing juveniles have travelled south-west onto shelf habitats of the Scotia Arc (Fig 7). The Antarctic Peninsula experiences annual sea ice cover, and the association of krill furcilia and juveniles to sea ice has been well documented [28,74–78]. The roles of sea ice for krill is an issue of active study [41,66,67] but the prevailing drift, both with ice and currents, transports larvae in a general north-easterly direction towards the Scotia Sea [79]. To counter this, it could be that the juveniles migrate back to the southern Scotia Arc where they concentrate during the summer. This possibility is in line with previous suggestions of horizontal migrations of krill throughout the season, for example, the concept of an offshore spawning migration [80] and inshore migrations to overwinter [49,81]. Contrasting early and late season bathymetric distributions of juveniles and adult male and female krill (Fig 6) provide further support for the concept of an active migration.

In contrast to all of the other life stages, the adult krill (males and females in both the early and later season) are much more ubiquitous throughout the study area. The very highest densities are found in habitats with very deep water (>4000) and relatively warm temperatures (2°C-4°C) that are characteristic of the north-eastern Scotia Sea. Adult krill can maintain swimming speeds of 10-15cms^-1^ [82] and advection must clearly play a strong role in governing their distribution [83,84]. However, the ability of krill to swim may allow them to move perpendicularly to the dominant flows [18,85,86]. This ability to influence their destination, in combination with a multi-year adult lifespan that allows time for dispersal, could explain such a broad distribution of the adult stages of krill.

### Wider implications

Notwithstanding our uncertainty over the driving mechanisms, our study shows that life stages of krill are partitioned between a range of habitats within their population centre in the south-west Atlantic sector. For a krill to reach adulthood, these different habitats must be utilised sequentially over the course of their development. Our study emphasises the importance of the tip of the Antarctic Peninsula to krill, being the only location where we find high densities of eggs, adults and juveniles, and where the high densities of calyptopes and furcilia could originate from.

The localised nature of krill spawning and nursery is also evidenced by the composite maps from the *Discovery Investigations* in the 1920s and 1930s [16]. Marr’s [16] multi-season composite bubble plots also show eggs concentrated at the tip of the Antarctic Peninsula, with high densities of calyptopes and furcilia in the middle of the Scotia Sea. However, recent work [87] has shown that for post-larvae, their centre of distribution in this sector has contracted southward by about 440 km between the *Discovery* era and the 1996–2016 period, commensurate with the substantial warming observed in this sector over that period [40]. Therefore, while the temperature-based distribution of the life stages may be responding as the climate of the sector changes, the basic concept of hotspots of spawning and early-stage nursery appears to hold both for the *Discovery* and the modern era.

This localised nature of successful spawning is important in the context of the krill fishing industry. Since the mid-1990s almost all krill fishing in the Southern Ocean has taken place in the south-west Atlantic, particularly around South Georgia, the South Orkneys and the South Shetland Islands and, more recently, in the Bransfield Strait [88–90]. The overlap of areas targeted by the fishery and the location of high adult densities is not surprising. However the presence of eggs in fished locations and the short time between spawning and hatch (~7 days at 0.5 ^o^C [53]), suggests that the fishery also overlaps with spawning sites. An objective of krill fishery management is to maintain stable recruitment of the target stock [91]. This objective might be harder to achieve if fishing intensity at spawning sites increases due either to an overall increase in fishing effort or a concentration of effort in these sites. CCAMLR aims to develop a finer scale management approach than the large subarea catch limits shown in Fig 1 [89]. Information on the location of spawning, such as that provided here, should be considered in the development of this approach to help minimise fishery impacts on recruitment.

Our study adds to the evidence that within the south-west Atlantic sector there are relatively localised hotspots of activity, including krill spawning and early stage development, foraging of land-based predators, fishing, tourism and scientific research [90–93]. We have identified the Southern Scotia Arc as one such hotspot for the krill life cycle. This area is also a focus for many human activities, increasing the potential for anthropogenic impacts on the ecosystem. The region is also undergoing long-term climatic warming and krill distribution and abundance appear to be changing in response [87]. Given the need to understand how krill will respond to future change, we hope that our data and maps describing the key areas for life-cycle completion form a baseline for future modelling initiatives.

## Supporting information

S1 Table Source of larval krill data, arranged with each row of data corresponding to a single research cruise. The complete larval database was larger than this, but the records listed here correspond to the screened subset of data that were used for plotting the distributions. References provided give more details on the specific research cruises. For a breakdown of the larval stages analysed on each cruise please see S2 Table.
(DOCX)

S2 Table The number of stations for each of the life stages is shown for each of the 41 seasons within the study period. In brackets next to this number is the percentage of those stations at which Antarctic krill were present. This table provides a breakdown of the stations into early and late season.
(DOCX)

S3 Table The data in the table below were used to determine the abundance of juvenile and adult (male and female) krill in both the early and late season. Blank cells signify no data
grid_1x2_ID: identifying numbers for each of the 1 degree of latitude by 2 degrees of longitude cells labelled in S1 Fig.
ave_frac15-30_earlyseason: The percentage of the catches from the grid cell that was 15-30mm in length from 1 October– 31 December of a season.
ave_frac15-30_lateseason: The percentage of the catches in the grid cell that was 15-30mm in length from 1 January–April 30 of a season.
>30mm_no_female: The number of measured krill in the catches within the grid cell that were >30mm in length and female.
>30mm_no_male: The equivalent number of measured krill in the catches that were >30mm in length and male.
ratio: The ratio of females to males for the grid cell.
(DOCX)

S4 Table The larval densities (egg, naup&meta, calyptope, furcilia) are those used for the larval maps. The postlarval densities were divided into juvenile (i.e. 15–30 mm, and the adults (>30mm) into male or female krill. These values were obtained from S2 Table. Environmental data for each of the grid cells is also in this table. This was used to create the depth histograms (Fig 5) and niche tables (Fig 6). Blank cells contain no data
grid_1x2_ID: identifying numbers for each of the 1 degree of latitude by 2 degrees of longitude grid cells.
egg_density: The average density of eggs (no. m^-2^) for each of the grid cells. There are only larval data for the late season.
naup&meta_density: The average density (no. m^-2^) of nauplii and metanauplii / m2 for each of the grid squares. There are only larval data for the late season.
caly_density: The average density (no. m^-2^) of calyptope for each of the grid squares. There are only larval data for the late season.
furc_density: The average density (no. m^-2^) of furcilia for each of the grid squares. There are only larval data for the late season.
adult_density_early: The density (no. m^-2^) of postlarval (>30mm) krill for each of the grid squares from 1 October– 31 December of a season.
adult_density_late: The average density (no. m^-2^) of eggs for each of the grid squares from 1 January–April 30, i.e. late season.
depth: Ocean bathymetry was sourced from the GEBCO data series. These data were used to create isobaths and to derive mean water depth for each of the grid cells.
SST: Climatological February mean sea surface temperature calculated as described in the main text.
(DOCX)

S5 Table This table contains the numbers of stations behind all of the density and length-frequency data. This information was used in the construction of Fig 6. Blank cells contain no data.
(DOCX)

S1 Fig The map shows the identifying numbers for each of the 1^o^ of latitude by 2 ^o^ of longitude grid cells in our south-west Atlantic study area. It is the same map as the main text Fig 1 which presents the latitude and longitudes of the map domain, allowing re-plotting and re-analyses of these data if necessary.
S3–S5 Tables link to these labelled cells, presenting the data extracted from KRILLBASE databases on krill post-larval and larval abundance and post-larval length frequency and plotted on a 1 degree latitude by 2 degree longitude grid. It thus contains the source data used for the construction of all the figures in the paper.
(EPS)
