# Supplementary material for: Habitat partitioning in Antarctic krill: Spawning hotspots and nursery areas
Source: PLoS One. 2019 Jul 24;14(7):e0219325. doi: 10.1371/journal.pone.0219325 (PMC6655634; doi:10.1371/journal.pone.0219325)
Supplement: S1 Table — The complete larval database was larger than this, but the records listed here correspond to the screened subset of data that were used for plotting the distributions. References provided give more details on the specific research cruises. For a breakdown of the larval stages analysed on each cruise please see S2 Table. (DOCX) [file pone.0219325.s001.docx]

| ***Year*** | **Month** | **Net type** | **Net depth**  **(m)** | **No. of**  **Stns.** | **Survey**  **(Reference)** | **Source of data** |
| --- | --- | --- | --- | --- | --- | --- |
| *1976* | 2,4 | RMT1*, Bongo | 0-200 | 128 | German SW Atlantic cruises  [1] | Transcribed by one of us (AA) from figures and tables of this reference |
| *1977* | 1 | 0.8 m Juday | 0-200  (mainly) | 13 | Norwegian Bouvet/Weddell Survey  [3] | Data were used in Fig. 2.5 in [2] and were provided by Siegel |
| *1979* | 2 | 1 m Nansen | 0-300 | 17 | Norwegian Weddell Sea Survey  [4] | Data were used in Fig. 2.5 in [2] and were provided by Siegel |
| *1980* | 1,2 | Bongo | 0-150  (variable) | 57 | German *Polarsirke*l cruise  [5] | Data were used in Fig. 2.5 in [2] and were provided by Siegel |
| *1981* | 1-3 | RMT1*  100 µm Juday  200 µm Nansen | 0-200  0-200  0-500/ 1000 | 307 | International FIBEX surveys (Russia, Argentina, Poland, Germany, Chile) across SW Atlantic | Data were used in Fig. 2.5 in [2] and were provided by Siegel Russian data transcribed by AA from [6] |
| *1982* | 2-3 | RMT1*  200 µm Nansen | 0-1000**  (variable) | 118 | German leg of *John Biscoe* Cruise [7] | Transcribed by one of us (AA) from figures and tables of this reference |
| *1985* | 1-3? | RMT 1? | 0-200 | 136 | German *Walter Herwig* cruise | Data were used in Fig. 2.5 in [2] and were provided by Siegel |
| *1987* | 1-3 | 330 µm 1m ring  net | 0-200**  0-1000** | 10 | US *Polar Duke* Cruise  [8] | Transcribed by one of us (AA) from figures and tables of this reference |
| *1989* | 1-2 | RMT1* | 0-250** | 1 | German Survey (reported in Ber. zur Polarforsch. 68 Ant-VII/4) | Data were used in Fig. 2.5 in [2] and were provided by Siegel |
| *1996* | 1 | 200 µm Bongo | 0-200 | 21 | British Antarctic Survey *James Clark Ross* Cruise  [9] | Obtained from BAS Polar Data Centre  <https://data.bas.ac.uk/> |
| *1997* | 1 | 200 µm Bongo | 0-200 | 1 | British Antarctic Survey *James Clark Ross* Cruise [9] | Obtained from BAS Polar Data Centre  <https://data.bas.ac.uk/> |
| *1998* | 1 | 200 µm Bongo | 0-200 | 8 | British Antarctic Survey *James Clark Ross* Cruise [9] | Obtained from BAS Polar Data Centre  <https://data.bas.ac.uk/> |
| *2000* | 1-2 | RMT1* | 0-200 | 157 | CCAMLR 2000 Synoptic Survey (Japan, Russia, USA and UK vessels)  [10] | Data were used in Fig. 2.5 in [2] and were provided by Siegel |
| *2001* | 4-5 | 300 µm Bongo | 0-300 | 13 | German *Polarstern* Cruise to Maguerite Bay  [11] | Original data provided by Pakhmov |
| *2001* | 1 | 200 µm Bongo | 0-200 | 8 | British Antarctic Survey *James Clark Ross Cruise*  [12] | Obtained from BAS Polar Data Centre  <https://data.bas.ac.uk/> |
| *2001* | 5 | 335 µm MOCNESS | 0-500**  (variable) | 20 | USA (Southern Ocean GLOBEC cruises)  [13] | Obtained from GLOBEC database  [www.globec.org](http://www.globec.org) |
| *2002* | 4-5 | 335 µm MOCNESS | 0-500**  (variable) | 20 | USA (Southern Ocean GLOBEC cruises)  [13] | Obtained from GLOBEC database  [www.globec.org](http://www.globec.org) |
| *2002* | 1,2 | 200 µm Bongo | 0-200 | 58 | British Antarctic Survey *James Clark Ross Cruise*  [12] | Obtained from BAS Polar Data Centre  <https://data.bas.ac.uk/> |
| *2003* | 1,2 | 200 µm Bongo | 0-200 | 61 | British Antarctic Survey *James Clark Ross* Cruise  [14] | Obtained from BAS Polar Data Centre  <https://data.bas.ac.uk/> |
| *2004* | 4 | RMT 1* | 0-200 | 92 | German *Polarstern* LAKRIS cruise  [15] | Data were used in Fig. 2.5 in [2] and were provided by Siegel |
| *2004* | 3 | 200 µm Bongo | 0-200 |  | British Antarctic Survey *James Clark Ross* Cruise  [16] | Obtained from BAS Polar Data Centre  <https://data.bas.ac.uk/> |
| *2005* | 1 | 200 µm Bongo | 0-200 |  | British Antarctic Survey *James Clark Ross* Cruise  [16] | Obtained from BAS Polar Data Centre  <https://data.bas.ac.uk/> |
| *2008* | 1,2 | Bongos  200 µm LHPR | 0-400  0-1000** | 67 | British Antarctic Survey *James Clark Ross* Cruise  [17] | Obtained from BAS Polar Data Centre  <https://data.bas.ac.uk/> |
| *2009* | 3,4 | Bongos  200 µm LHPR | 0-400  0-1000** | 70 | British Antarctic Survey *James Clark Ross* Cruise  [17] | Obtained from BAS Polar Data Centre  <https://data.bas.ac.uk/> |
| *2011* | 1 | RMT1* | 0-200 | 94 | German *Polarstern* Cruise  [15] | Data were used in Fig. 2.5 in [2] and were provided by Siegel |
| *2011* | 1 | 505 µm IKMT | 0-170 | 81 | US AMLR cruise  [15] | Data were used in Fig. 2.5 in [2] and were provided by Siegel |

* Typical mesh size of RMT 1 net is 330 µm

**Stratified hauls, here combined to provide total densities under 1 m^2^ in whole sampled water column

**References for S1 Table**

1. Hempel I, Hempel G. Larval krill (*Euphausia superba*) in the plankton and neuston samples of the German Antarctic Expedition 1975/1976.Meerserfoschung. 1978;26:207-216.
2. Siegel V, Watkins J. Distribution, biomass and demography of Antarctic krill, *Euphausia superba*. In: Siegel V, editor. Biology and ecology of Antarctic krill. Springer; 2016. p. 21–100.
3. Fevolden SE. Investigations on krill (Euphausiacea) sampled during the Norwegian Antarctic Research Expedition 1976/77. Sarsia. 1979;64:189-198.
4. Fevolden SE. Krill off Bouvetöya and in the southern Weddell Sea with a description of larval stages of *Euphausia crystallorophias*. Sarsia. 1980;65:149-162.
5. Hempel I, Hempel G. Distribution of euphausiid larvae in the southern Weddell Sea. Meeresforsch. 1982;29:253–266.
6. Makarov RR, Menshenina LL. Larvae of euphausiids off Queen Maud Land. Polar Biol. 1992;11:515-523.
7. Marschall S, Mizdalski E. Euphausiid larvae in plankton samples from the vicinity of the Antarctic Peninsula, February 1982. Ber. zur Polarforsch.1985;21: 5-47.
8. Marin VH, Brinton E, Huntley M. Depth relationships of *Euphausia superba* eggs, larvae and adults near the Antarctic Peninsula, 1986-87. Deep-Sea Res. 1991;38:1241-1249.
9. Ward P, Whitehouse M, Brandon M, Shreeve R, Wood-Walker R. Mesozooplankton community structure across the Antarctic Circumpolar Current to the north of South Georgia: Southern Ocean. Mar Biol. 2003;143:121-130.
10. Siegel V, Kawaguchi S, Ward P, Litvinov F, Sushin V, Loeb V, Watkins J. Krill demography and large-scale distribution in the southwest Atlantic during January/Februray 2000. Deep-Sea Res II. 2004;51:1253-1273.
11. Pakhomov EA, Atkinson A, Meyer B, Oettl B, Bathmann U. Daily rations and growth of larval *Euphausia superba* in the Eastern Bellingshausen Sea during austral autumn. Deep-Sea Res. 2004;II 51: 2185-2198.
12. Tarling GA, Cuzin-Roudy J, Thorpe SE, Shreeve RS, Ward P, Murphy EJ. Recruitment of Antarctic krill *Euphausia superba* in the South Georgia region: adult fecundity and the fate of larvae Mar. Ecol. Progr. Ser. 2007;331:161-179.
13. Wiebe PH, Ashjian CJ, Lawsonj GL, Piñones A, Copley NJ. Horizontal and vertical distribution of euphausiid species on the Western Antarctic Peninsula U.S. GLOBEC Southern Ocean study site. Deep-Sea Res II. 2011;58:1630-1651.
14. Ward P, Shreeve R, Atkinson A, Korb R, Whitehouse M, Thorpe S, Pond D, Cunnigham N. Plankton community structure and variability in the Scotia Sea: austral summer 2003. Mar. Ecol. Progr. Ser. 2006;309:75-91.
15. Siegel V, Reiss CS, Dietrich KS, Haraldsson M, Rohardt G. Distribution and abundance of Antarctic krill (*Euphausia superba*) along the Antarctic Peninsula. Deep-Sea Res 1. 2013;77:63-74
16. Ward P, Meredith MP, Whitehouse MJ, Rothery P. The summertime plankton community at South Georgia (Southern Ocean): comparing the historical (1926/1927) and modern (post1995) records. Progr. Oceanogr. 2008;78:241-256.
17. Ward P, Atkinson A, Tarling G. Mesozooplankton community structure and variability in the Scotia Sea: a seasonal comparison. Deep-Sea Res. 2012;59/60:78-93.

.
