# Supplementary material for: Habitat partitioning in Antarctic krill: Spawning hotspots and nursery areas
Source: PLoS One. 2019 Jul 24;14(7):e0219325. doi: 10.1371/journal.pone.0219325 (PMC6655634; doi:10.1371/journal.pone.0219325)
Supplement: S2 Table — In brackets next to this number is the percentage of those stations at which Antarctic krill were present. This table provides a breakdown of the stations into early and late season. (DOCX) [file pone.0219325.s002.docx]

| Number of stations (percentage presence) | | | | | | | | | | |
| --- | --- | --- | --- | --- | --- | --- | --- | --- | --- | --- |
| SEASON | larval_data | | | | length-frequency_data | | | | abundance_data | |
|  | Eggs | Nauplii | Calyptopes | Furcilia | 15-30mm_early | >30mm_early | 15-30mm_late | >30mm_late | early | late |
| 1976 |  |  | 128(41) | 127(21) |  |  | 60(93) | 60(100) | 60(85) | 133(78) |
| 1977 |  |  | 13(0) | 13(0) |  |  |  |  |  |  |
| 1978 |  |  |  |  | 39(85) | 39(95) | 107(68) | 107(97) | 51(86) | 172(97) |
| 1979 |  |  | 17(82) | 17(0) |  |  | 8(75) | 8(100) |  |  |
| 1980 |  |  | 57(24) | 57(2) | 3(100) | 3(100) | 120(3) | 120(100) |  | 52(4) |
| 1981 |  | 79(3) | 307(68) | 307(39) | 24(96) | 24(91) | 179(59) | 179(100) | 23(100) | 169(53) |
| 1982 | 118(73) | 118(33) | 118(76) | 118(23) | 32(97) | 32(97) | 337(85) | 337(91) | 191(45) | 59(86) |
| 1983 |  |  |  |  |  |  | 30(93) | 30(97) |  | 3(100) |
| 1984 |  |  |  |  | 162(98) | 162(80) | 195(46) | 195(96) | 150(85) | 192(55) |
| 1985 |  |  |  | 136(51) | 175(45) | 175(98) | 274(15) | 274(100) | 197(67) | 230(84) |
| 1986 |  |  |  |  |  |  | 92(29) | 92(92) |  | 22(86) |
| 1987 | 1(100) | 10(30) | 10(90) | 10(60) | 6(67) | 6(83) |  |  |  | 18(89) |
| 1988 |  |  |  |  | 76(82) | 76(96) | 85(65) | 85(100) | 79(97) | 131(75) |
| 1989 |  |  | 1(100) | 1(0) | 20(95) | 20(90) | 2(100) | 2(100) | 21(95) | 11(45) |
| 1990 |  |  |  |  | 21(24) | 21(100) | 76(30) | 76(100) | 24(83) | 139(55) |
| 1991 |  |  |  |  | 14(86) | 14(100) | 107(36) | 107(97) | 19(0) | 80(43) |
| 1992 |  |  |  |  |  |  | 110(66) | 110(98) |  | 162(89) |
| 1993 |  |  |  |  |  |  | 136(29) | 136(99) |  | 211(86) |
| 1994 |  |  |  |  |  |  | 107(27) | 107(94) | 3(100) | 272(75) |
| 1995 |  |  |  |  | 74(53) | 74(96) | 114(17) | 114(100) | 75(99) | 255(79) |
| 1996 | 21(0) | 21(0) | 21(24) | 21(0) |  |  | 163(67) | 163(98) | 72(29) | 322(81) |
| 1997 | 10(0) | 1(0) | 1(0) | 1(0) | 100(88) | 100(99) | 81(32) | 81(96) | 130(82) | 194(90) |
| 1998 |  |  |  |  | 3(67) | 3(100) | 140(72) | 140(97) |  | 321(84) |
| 1999 | 8(0) | 8(38) | 8(0) | 8(0) |  |  | 57(2) | 57(100) | 29(21) | 241(70) |
| 2000 | 157(14) | 36(6) | 157(48) | 157(9) | 1(100) | 1(100) | 265(33) | 265(99) |  | 261(75) |
| 2001 | 12(0) | 12(0) | 41(41) | 41(71) | 21(90) | 21(100) | 181(45) | 181(98) | 14(64) | 369(80) |
| 2002 | 59(0) | 59(5) | 78(44) | 77(21) |  |  | 120(54) | 120(91) |  | 313(61) |
| 2003 | 61(15) | 61(18) | 61(39) | 61(2) |  |  | 108(71) | 108(95) |  | 306(78) |
| 2004 | 19(0) | 19(0) | 111(54) | 111(42) |  |  | 95(21) | 95(99) |  | 271(75) |
| 2005 | 37(3) | 37(3) | 37(16) | 37(3) | 1(0) | 1(100) | 89(11) | 89(99) | 20(0) | 290(74) |
| 2006 |  |  |  |  |  |  | 39(18) | 39(100) |  | 159(89) |
| 2007 |  |  |  |  |  |  | 46(87) | 46(100) | 28(21) | 170(95) |
| 2008 |  | 56(0) | 67(28) | 67(1) |  |  | 22(36) | 22(100) |  | 307(79) |
| 2009 |  | 61(0) | 70(59) | 70(29) |  |  | 9(0) | 9(100) |  | 197(68) |
| 2010 |  |  |  |  | 9(67) | 9(100) |  |  |  | 107(79) |
| 2011 |  | 120(2) | 175(54) | 174(10) | 6(17) | 6(100) | 10(80) | 10(100) |  | 120(100) |
| 2012 |  |  |  |  |  |  | 9(33) | 9(100) |  | 41(95) |
| 2013 |  |  |  |  | 8(0) | 8(100) |  |  |  | 42(100) |
| 2014 |  |  |  |  | 10(90) | 10(100) |  |  |  | 33(100) |
| 2015 |  |  |  |  | 7(71) | 7(100) |  |  |  | 37(92) |
| 2016 |  |  |  |  | 9(67) | 9(100) |  |  |  | 29(100) |
