# Supplementary material for: Habitat partitioning in Antarctic krill: Spawning hotspots and nursery areas
Source: PLoS One. 2019 Jul 24;14(7):e0219325. doi: 10.1371/journal.pone.0219325 (PMC6655634; doi:10.1371/journal.pone.0219325)
Supplement: S3 Table — Blank cells signify no data grid_1x2_ID: identifying numbers for each of the 1 degree of latitude by 2 degrees of longitude cells labelled in S1 Fig. ave_frac15-30_earlyseason: The percentage of the catches from the grid cell that was 15-30mm in length from 1 October– 31 December of a season. ave_frac15-30_lateseason: The percentage of the catches in the grid cell that was 15-30mm in length from 1 January–April 30 of a season. >30mm_no_female: The number of measured krill in the catches within the grid cell that were >30mm in length and female. >30mm_no_male: The equivalent number of measured krill in the catches that were >30mm in length and male. ratio: The ratio of females to males for the grid cell. (DOCX) [file pone.0219325.s003.docx]

| grid_1x2_ID | ave_frac15-30_earlyseason | ave_frac15-30_lateseason | >30mm_no_female | >30mm_no_male | ratio |
| --- | --- | --- | --- | --- | --- |
| 2229 |  | 0 | 5 | 4 | 56:44 |
| 2415 | 20.68965517 |  | 34 | 24 | 59:41 |
| 2596 | 85.71428571 | 10.52631579 | 15 | 6 | 71:29 |
| 2776 | 0 |  | 0 | 14 | 0:100 |
| 2777 |  | 91.84861718 | 28 | 39 | 42:58 |
| 2958 | 0 |  | 0 | 1 | 0:100 |
| 2959 | 82.60869565 | 100 | 13 | 11 | 54:46 |
| 3113 |  | 0 | 30 | 12 | 71:29 |
| 3139 |  | 39.87068966 | 159 | 124 | 56:44 |
| 3140 | 91.19850187 | 16.47058824 | 249 | 233 | 52:48 |
| 3291 |  | 5.555555556 | 6 | 11 | 35:65 |
| 3319 |  | 98.22747415 | 8 | 9 | 47:53 |
| 3680 |  | 16.66666667 | 2 | 4 | 33:67 |
| 3833 |  | 32.25806452 | 4 | 17 | 19:81 |
| 3835 |  | 0 | 2 | 4 | 33:67 |
| 4013 |  | 2.469135802 | 61 | 19 | 76:24 |
| 4014 | 60.34482759 | 0 | 17 | 35 | 33:67 |
| 4015 |  | 22.18468468 | 850 | 594 | 59:41 |
| 4016 |  | 16.81675393 | 83 | 431 | 16:84 |
| 4029 |  | 0 | 1 | 0 | 100:0 |
| 4033 |  | 40 | 1 | 2 | 33:67 |
| 4036 |  | 3.125 | 19 | 12 | 61:39 |
| 4192 |  | 0 | 52 | 82 | 39:61 |
| 4193 |  | 10.95890411 | 92 | 117 | 44:56 |
| 4194 |  | 28.94736842 | 35 | 47 | 43:57 |
| 4195 | 77.72435897 | 58.16039839 | 1100 | 1619 | 40:60 |
| 4196 |  | 5.266705613 | 683 | 560 | 55:45 |
| 4205 |  | 0 | 6 | 7 | 46:54 |
| 4373 |  | 0 | 3 | 5 | 38:63 |
| 4374 | 14.28571429 | 0 | 31 | 30 | 51:49 |
| 4375 | 90.66666667 | 0 | 204 | 123 | 62:38 |
| 4376 | 81.52173913 | 18.52791878 | 999 | 1214 | 45:55 |
| 4384 |  | 57.14285714 | 2 | 1 | 67:33 |
| 4385 |  | 78.51099831 | 2 | 80 | 2:98 |
| 4386 |  | 43.37011904 | 171 | 476 | 26:74 |
| 4554 |  | 0 | 1 | 4 | 20:80 |
| 4555 | 0 | 0.549450549 | 136 | 201 | 40:60 |
| 4556 | 0 | 30.23255814 | 226 | 189 | 54:46 |
| 4557 | 9.916970332 | 28.77526754 | 2142 | 2051 | 51:49 |
| 4558 | 15.19607843 | 23.45996995 | 1145 | 1606 | 42:58 |
| 4559 |  | 43.47081401 | 174 | 222 | 44:56 |
| 4562 |  | 1.763224181 | 788 | 1136 | 41:59 |
| 4563 |  | 13.63636364 | 7 | 12 | 37:63 |
| 4564 |  | 4.230769231 | 112 | 139 | 45:55 |
| 4567 |  | 16.56050955 | 89 | 29 | 75:25 |
| 4568 |  | 19.51219512 | 28 | 40 | 41:59 |
| 4735 | 0 | 0 | 24 | 47 | 34:66 |
| 4736 | 0 | 0 | 95 | 55 | 63:37 |
| 4737 | 15.76763485 | 14.89361702 | 904 | 731 | 55:45 |
| 4738 | 7.291333547 | 9.758501725 | 2239 | 2142 | 51:49 |
| 4739 | 43.98066094 | 23.13708451 | 3908 | 4547 | 46:54 |
| 4740 | 29.58953541 | 23.38235294 | 1922 | 2480 | 44:56 |
| 4741 | 58.71369295 | 35.4076218 | 776 | 704 | 52:48 |
| 4742 | 25 | 10.4816825 | 1133 | 1302 | 47:53 |
| 4743 |  | 12.07207207 | 254 | 201 | 56:44 |
| 4744 |  | 91.98498914 | 2 | 0 | 100:0 |
| 4745 |  | 88.27886889 | 10 | 4 | 71:29 |
| 4746 | 95.04950495 |  | 13 | 14 | 48:52 |
| 4916 |  | 0 | 220 | 185 | 54:46 |
| 4917 | 50 | 0 | 8 | 2 | 80:20 |
| 4918 | 35.98820059 | 0.220264317 | 213 | 465 | 31:69 |
| 4919 | 26.16179002 | 6.095952936 | 3584 | 3465 | 51:49 |
| 4920 | 46.35274976 | 31.231474 | 1471 | 1582 | 48:52 |
| 4921 | 48.28193041 | 25.12067914 | 3628 | 3661 | 50:50 |
| 4922 | 56.61132904 | 20.57577086 | 3221 | 3444 | 48:52 |
| 4923 | 97.78481013 | 12.72777594 | 1827 | 982 | 65:35 |
| 4924 |  | 10.69518717 | 1557 | 416 | 79:21 |
| 4925 | 41.30434783 | 51.35967245 | 310 | 51 | 86:14 |
| 4926 | 17.78455285 |  | 507 | 454 | 53:47 |
| 4927 |  | 0 | 2 | 7 | 22:78 |
| 4930 | 100 |  |  |  |  |
| 4931 | 92.5 |  | 0 | 3 | 0:100 |
| 4939 |  | 26.41196013 | 401 | 402 | 50:50 |
| 4940 |  | 8.208955224 |  |  |  |
| 5098 | 0 | 0 | 6 | 39 | 13:87 |
| 5099 | 4.010025063 | 0.620567376 | 664 | 513 | 56:44 |
| 5100 | 29.04911181 | 1.743638077 | 561 | 800 | 41:59 |
| 5101 | 30.07606548 | 8.101110788 | 6936 | 6368 | 52:48 |
| 5102 | 41.85098084 | 15.60281599 | 10295 | 7933 | 56:44 |
| 5103 | 21.95914198 | 12.60983505 | 2606 | 1673 | 61:39 |
| 5104 |  | 0.787401575 | 646 | 106 | 86:14 |
| 5105 | 21.08013937 | 0 | 416 | 1218 | 25:75 |
| 5106 | 5.637583893 | 2.638522427 | 405 | 459 | 47:53 |
| 5107 |  | 61.50234742 | 2 | 27 | 7:93 |
| 5108 |  | 0 | 0 | 6 | 0:100 |
| 5109 |  | 0 |  |  |  |
| 5110 | 100 |  |  |  |  |
| 5111 | 95.46322195 | 13.03418803 | 14 | 37 | 27:73 |
| 5112 |  | 0 |  |  |  |
| 5114 |  | 9.736842105 | 16 | 49 | 25:75 |
| 5277 |  | 0.436681223 | 82 | 143 | 36:64 |
| 5278 | 100 |  |  |  |  |
| 5279 |  | 0 | 140 | 190 | 42:58 |
| 5280 | 3.378684807 | 0.20746888 | 365 | 279 | 57:43 |
| 5281 | 11.39306965 | 8.547557841 | 3414 | 2482 | 58:42 |
| 5282 | 17.0918832 | 18.36955078 | 17307 | 13223 | 57:43 |
| 5283 | 13.00365041 | 2.623021895 | 820 | 784 | 51:49 |
| 5284 | 63.98809774 | 0 | 162 | 402 | 29:71 |
| 5285 | 9.759976865 | 1.798279906 | 225 | 210 | 52:48 |
| 5286 | 20.40956439 | 1.279554937 | 788 | 383 | 67:33 |
| 5287 | 0 | 1.921165949 | 436 | 315 | 58:42 |
| 5288 | 3.921568627 | 0.762016413 | 226 | 142 | 61:39 |
| 5289 | 34.89975208 | 0 | 39 | 45 | 46:54 |
| 5290 | 67.29842891 |  | 43 | 22 | 66:34 |
| 5291 | 100 |  |  |  |  |
| 5292 |  | 10.2484472 | 0 | 2 | 0:100 |
| 5294 |  | 0 |  |  |  |
| 5295 |  | 50 |  |  |  |
| 5296 |  | 84.21052632 | 27 | 15 | 64:36 |
| 5297 |  | 8.333333333 | 46 | 31 | 60:40 |
| 5458 |  | 6.818181818 | 847 | 1133 | 43:57 |
| 5461 | 3.583167331 | 1.941747573 | 159 | 86 | 65:35 |
| 5462 | 0.684931507 | 0 | 403 | 409 | 50:50 |
| 5463 | 4.183266932 | 0.343642612 | 275 | 145 | 65:35 |
| 5464 | 59.47136564 | 0.350877193 | 214 | 254 | 46:54 |
| 5465 | 16.60952381 | 1.462166443 | 4833 | 2725 | 64:36 |
| 5466 | 11.67400881 | 0.183234082 | 558 | 477 | 54:46 |
| 5467 | 0 | 0.058326043 | 459 | 1049 | 30:70 |
| 5468 | 0 | 1.014198783 | 2637 | 1626 | 62:38 |
| 5469 | 4.379562044 | 0.787401575 |  |  |  |
| 5470 | 83.90221665 | 1.992031873 | 75 | 109 | 41:59 |
| 5471 | 70.58341428 | 2.247191011 | 113 | 114 | 50:50 |
| 5472 |  | 9.901960784 | 261 | 112 | 70:30 |
| 5473 |  | 29.37293729 | 14 | 64 | 18:82 |
| 5474 |  | 1.893287435 |  |  |  |
| 5477 |  | 66.08391608 | 93 | 43 | 68:32 |
| 5478 |  | 50.94339623 | 18 | 8 | 69:31 |
| 5638 | 9.487951807 | 10.82014912 |  |  |  |
| 5644 |  | 0 | 72 | 18 | 80:20 |
| 5645 | 2.884615385 | 0 | 10 | 7 | 59:41 |
| 5646 | 26.63185379 | 1.680672269 | 157 | 76 | 67:33 |
| 5647 | 14.25 | 0 | 12 | 10 | 55:45 |
| 5648 |  | 0.145560408 | 251 | 231 | 52:48 |
| 5649 | 1.428571429 | 0 | 68 | 52 | 57:43 |
| 5650 | 100 |  | 2 | 0 | 100:0 |
| 5651 | 59.65417867 | 4.972375691 | 41 | 76 | 35:65 |
| 5652 |  | 7.225433526 |  |  |  |
| 5653 |  | 10.13071895 | 0 | 6 | 0:100 |
| 5654 |  | 4.306220096 |  |  |  |
| 5655 |  | 55.66600398 | 117 | 94 | 55:45 |
| 5656 |  | 58.21917808 | 224 | 187 | 55:45 |
| 5657 |  | 32.82051282 | 165 | 126 | 57:43 |
| 5658 |  | 16.26506024 | 81 | 58 | 58:42 |
| 5824 |  | 0 | 5 | 3 | 63:38 |
| 5826 | 100 |  |  |  |  |
| 5827 |  | 4.255319149 | 50 | 61 | 45:55 |
| 5828 |  | 0 | 40 | 72 | 36:64 |
| 5829 |  | 0 | 2 | 64 | 3:97 |
| 5831 |  | 18.46153846 | 134 | 26 | 84:16 |
| 5832 |  | 4.255319149 | 0 | 36 | 0:100 |
| 5833 | 17 |  |  |  |  |
| 5834 | 30.24390244 | 13.51351351 | 83 | 44 | 65:35 |
| 5835 |  | 18.55116903 | 818 | 817 | 50:50 |
| 5836 |  | 68.62068966 | 11 | 13 | 46:54 |
| 5838 |  | 0 | 63 | 50 | 56:44 |
| 6008 |  | 0 | 2 | 79 | 2:98 |
| 6009 |  | 0 | 66 | 50 | 57:43 |
| 6010 |  | 0 | 2 | 2 | 50:50 |
| 6011 |  | 0 |  |  |  |
| 6012 | 60.72796935 | 9.592061742 | 59 | 76 | 44:56 |
| 6013 |  | 31.39158576 | 102 | 70 | 59:41 |
| 6014 |  | 38.35376532 |  |  |  |
| 6016 |  | 3.361344538 | 46 | 69 | 40:60 |
| 6017 |  | 4.651162791 | 23 | 20 | 53:47 |
| 6189 |  | 100 |  |  |  |
| 6190 | 82.63736264 | 100 |  |  |  |
| 6191 | 64.0077821 | 13.25 | 20 | 74 | 21:79 |
| 6192 | 83 | 25.37313433 | 0 | 2 | 0:100 |
| 6193 | 92.53731343 | 2.5 | 4 | 1 | 80:20 |
| 6194 | 6 | 16.84210526 | 11 | 23 | 32:68 |
| 6195 |  | 0 | 34 | 36 | 49:51 |
| 6196 |  | 0.369685767 | 381 | 158 | 71:29 |
| 6197 |  | 0 | 1 | 2 | 33:67 |
| 6200 |  | 0 |  |  |  |
| 6368 |  | 0 | 42 | 68 | 38:62 |
| 6369 | 97.08737864 | 0 | 0 | 2 | 0:100 |
| 6370 | 10 | 0.138664202 | 1963 | 2114 | 48:52 |
| 6371 | 64.0904551 | 36.12108774 | 149 | 289 | 34:66 |
| 6372 | 30.47676524 | 37.06828436 | 912 | 1463 | 38:62 |
| 6373 | 79.91718427 | 22.40779402 | 65 | 208 | 24:76 |
| 6374 | 97.66666667 | 9.090909091 | 4 | 2 | 67:33 |
| 6375 |  | 3 | 61 | 36 | 63:37 |
| 6549 | 0 | 12.05673759 | 79 | 101 | 44:56 |
| 6550 | 12.3465288 | 13.46054667 | 3247 | 3350 | 49:51 |
| 6551 | 36.60518912 | 33.79861081 | 3926 | 5431 | 42:58 |
| 6552 | 56.34563456 | 45.41198502 | 113 | 172 | 40:60 |
| 6553 | 92.4205379 | 2 | 22 | 7 | 76:24 |
| 6554 |  | 5.925925926 | 18 | 48 | 27:73 |
| 6556 |  | 0 | 1 | 2 | 33:67 |
| 6729 |  | 13 | 101 | 97 | 51:49 |
| 6730 |  | 3.731343284 | 104 | 122 | 46:54 |
| 6731 | 55.22827688 | 4.347826087 | 168 | 125 | 57:43 |
| 6733 |  | 0 | 71 | 115 | 38:62 |
| 6736 |  | 0 | 4 | 1 | 80:20 |
| 6909 |  | 38.24362606 | 16 | 46 | 26:74 |
| 6910 |  | 0 | 0 | 2 | 0:100 |
| 6913 |  | 1.960784314 | 18 | 52 | 26:74 |
| 6915 |  | 0 | 5 | 4 | 56:44 |
