# Supplementary material for: Habitat partitioning in Antarctic krill: Spawning hotspots and nursery areas
Source: PLoS One. 2019 Jul 24;14(7):e0219325. doi: 10.1371/journal.pone.0219325 (PMC6655634; doi:10.1371/journal.pone.0219325)
Supplement: S4 Table — The postlarval densities were divided into juvenile (i.e. 15–30 mm, and the adults (>30mm) into male or female krill. These values were obtained from S2 Table. Environmental data for each of the grid cells is also in this table. This was used to create the depth histograms (Fig 5) and niche tables (Fig 6). Blank cells contain no data grid_1x2_ID: identifying numbers for each of the 1 degree of latitude by 2 degrees of longitude grid cells. egg_density: The average density of eggs (no. m-2) for each of the grid cells. There are only larval data for the late season. naup&meta_density: The average density (no. m-2) of nauplii and metanauplii / m2 for each of the grid squares. There are only larval data for the late season. caly_density: The average density (no. m-2) of calyptope for each of the grid squares. There are only larval data for the late season. furc_density: The average density (no. m-2) of furcilia for each of the grid squares. There are only larval data for the late season. adult_density_early: The density (no. m-2) of postlarval (>30mm) krill for each of the grid squares from 1 October– 31 December of a season. adult_density_late: The average density (no. m-2) of eggs for each of the grid squares from 1 January–April 30, i.e. late season. depth: Ocean bathymetry was sourced from the GEBCO data series. These data were used to create isobaths and to derive mean water depth for each of the grid cells. SST: Climatological February mean sea surface temperature calculated as described in the main text. (DOCX) [file pone.0219325.s004.docx]

| grid_1x2_ID | egg_density | naup&meta_density | caly_density | furc_density | adult_density_early | adult_density_late | depth | SST |
| --- | --- | --- | --- | --- | --- | --- | --- | --- |
| 2225 |  |  | 0 | 0 |  | 0 |  |  |
| 2226 |  |  | 0 | 0 |  | 0 | -185.17 | -1.6487 |
| 2227 |  |  | 0 | 0 |  | 0 | -297.06 | -1.6473 |
| 2228 |  |  | 0 | 0 |  | 0.8532 | -482.83 | -1.6465 |
| 2229 |  |  | 0 | 0 |  | 0 | -729.11 | -1.6265 |
| 2230 |  |  |  |  |  | 0 | -998.59 | -1.607 |
| 2231 |  |  | 2.365273001 | 0 |  | 0 | -1024.46 | -1.592 |
| 2232 |  |  | 6.369426752 | 0 |  | 0 | -573.73 | -1.7153 |
| 2233 |  |  |  |  |  |  | -290.82 | -1.676 |
| 2402 |  |  | 0 | 0 |  | 0 | -430.04 | -1.652 |
| 2403 |  |  | 0 | 0 |  | 0 | -345.64 | -1.591 |
| 2404 |  |  | 0 | 0 |  | 0 | -319.74 | -1.5857 |
| 2405 |  |  | 0.141509434 | 0 |  | 0.064285714 | -318.73 | -1.606 |
| 2406 |  |  | 0 | 0 |  | 0 | -302.02 | -1.628 |
| 2407 |  |  |  |  |  |  | -328.25 | -1.636 |
| 2408 |  |  |  |  |  |  | -382.61 | -1.6485 |
| 2409 |  |  |  |  |  |  | -467.63 | -1.6503 |
| 2410 |  |  |  |  |  |  | -644.50 | -1.6263 |
| 2411 |  |  |  |  |  |  | -803.87 | -1.5725 |
| 2412 |  |  |  |  |  |  | -861.47 | -1.5097 |
| 2413 |  |  | 1.167315175 | 0 |  | 0 | -629.14 | -1.5107 |
| 2414 |  |  | 23.26968974 | 0 |  | 0 | -387.05 | -1.8505 |
| 2415 |  |  |  |  |  | 0 | -348.26 | -1.358 |
| 2579 |  |  | 0 | 0 |  | 0 |  |  |
| 2580 |  |  | 0 | 0 |  | 0 | -507.14 | -1.491 |
| 2581 |  |  | 0 | 0 |  | 0 | -474.73 | -1.491 |
| 2582 |  |  | 0 | 0 |  |  | -445.47 | -1.6185 |
| 2583 |  |  |  |  |  |  | -474.47 | -1.6157 |
| 2584 |  |  |  |  |  |  | -482.08 | -1.612 |
| 2585 |  |  | 0 | 0 |  | 0 | -456.53 | -1.615 |
| 2586 |  |  |  |  |  |  | -366.23 | -1.613 |
| 2587 |  |  |  |  |  |  | -324.58 | -1.585 |
| 2588 |  |  |  |  |  |  | -351.79 | -1.594 |
| 2589 |  |  |  |  |  |  | -386.49 | -1.6117 |
| 2590 |  |  |  |  |  |  | -405.24 | -1.625 |
| 2591 |  |  |  |  |  |  | -460.34 | -1.608 |
| 2592 |  |  |  |  |  |  | -583.02 | -1.52 |
| 2593 |  |  |  |  |  |  | -673.64 | -1.3783 |
| 2594 |  |  |  |  |  |  | -558.85 | -1.27 |
| 2595 |  |  |  |  |  |  | -427.89 | -1.135 |
| 2596 |  |  | 27.7999651 | 0 |  | 0.994 | -358.20 | -1.223 |
| 2759 |  |  | 0 | 0 |  | 0 | -457.51 | -1.5853 |
| 2760 |  |  |  |  |  |  | -591.26 | -1.579 |
| 2761 |  |  |  |  |  |  | -527.29 | -1.5905 |
| 2762 |  |  |  |  |  |  | -456.53 | -1.6105 |
| 2763 |  |  |  |  |  |  | -415.91 | -1.6182 |
| 2764 |  |  |  |  |  |  | -398.15 | -1.622 |
| 2765 |  |  |  |  |  |  | -442.85 | -1.6245 |
| 2766 |  |  |  |  |  |  | -486.73 | -1.6227 |
| 2767 |  |  |  |  |  |  | -488.42 | -1.5998 |
| 2768 |  |  |  |  |  |  | -418.85 | -1.5685 |
| 2769 |  |  |  |  |  |  | -403.38 | -1.5528 |
| 2770 |  |  | 242.0382166 | 0 |  |  | -528.10 | -1.5507 |
| 2771 |  |  | 93.41825902 | 0 |  |  | -871.05 | -1.536 |
| 2772 |  |  | 945.8598726 | 0 |  |  | -1012.69 | -1.4823 |
| 2773 |  |  | 60.50955414 | 0 |  |  | -849.63 | -1.3653 |
| 2774 |  |  |  |  |  |  | -930.81 | -1.2245 |
| 2775 |  |  |  |  |  |  | -1334.36 | -1.0797 |
| 2776 |  |  |  |  |  |  | -1533.53 | -1.121 |
| 2777 |  |  | 0 | 0 |  | 0 | -907.09 | -1.3548 |
| 2778 |  |  |  |  |  |  | -179.06 | -1.485 |
| 2779 |  |  |  |  |  |  | -110.44 | -1.481 |
| 2940 |  |  |  |  |  |  | -511.83 | -1.5813 |
| 2941 |  |  |  |  |  |  | -523.18 | -1.59 |
| 2942 |  |  |  |  |  |  | -492.67 | -1.606 |
| 2943 |  |  |  |  |  |  | -434.54 | -1.618 |
| 2944 |  |  |  |  |  |  | -391.54 | -1.6207 |
| 2945 |  |  |  |  |  |  | -374.50 | -1.6225 |
| 2946 |  |  |  |  |  |  | -400.24 | -1.625 |
| 2947 |  |  |  |  |  |  | -589.22 | -1.6257 |
| 2948 |  |  |  |  |  |  | -969.95 | -1.6195 |
| 2949 |  |  |  |  |  |  | -1547.38 | -1.586 |
| 2950 |  |  | 70.06369427 | 0 |  |  | -2168.22 | -1.4993 |
| 2951 |  |  |  |  |  |  | -2663.80 | -1.4395 |
| 2952 |  |  | 0 | 0 |  |  | -2860.75 | -1.3827 |
| 2953 |  |  |  |  |  |  | -2832.45 | -1.2723 |
| 2954 |  |  |  |  |  |  | -2953.73 | -1.1535 |
| 2955 |  |  |  |  |  |  | -3130.68 | -1.0473 |
| 2956 |  |  |  |  |  |  | -3124.58 | -1.0457 |
| 2957 |  |  |  |  |  |  | -3152.75 | -1.146 |
| 2958 |  |  | 0 | 0 |  | 74.864 | -1827.42 | -1.2397 |
| 2959 |  |  | 0 | 0 |  |  | -922.67 | -1.261 |
| 3111 |  |  |  |  |  |  | -37.64 | -0.8753 |
| 3112 |  |  |  |  |  |  | -16.76 | -0.9033 |
| 3113 |  |  |  |  |  | 8.9725 | -10.46 | -0.9998 |
| 3120 |  |  |  |  |  |  | -290.94 | -1.559 |
| 3121 |  |  |  |  |  |  | -516.12 | -1.571 |
| 3122 |  |  |  |  |  |  | -550.49 | -1.5995 |
| 3123 |  |  |  |  |  |  | -629.21 | -1.626 |
| 3124 |  |  |  |  |  |  | -604.47 | -1.628 |
| 3125 |  |  |  |  |  |  | -539.69 | -1.6295 |
| 3126 |  |  |  |  |  |  | -982.95 | -1.631 |
| 3127 |  |  |  |  |  |  | -1617.97 | -1.633 |
| 3128 |  |  |  |  |  |  | -2213.74 | -1.641 |
| 3129 |  |  |  |  |  |  | -2763.38 | -1.629 |
| 3130 |  |  |  |  |  |  | -3454.13 | -1.534 |
| 3131 |  |  |  |  |  |  | -3485.65 | -1.4475 |
| 3132 |  |  |  |  |  |  | -3586.03 | -1.3687 |
| 3133 |  |  |  |  |  |  | -3754.39 | -1.241 |
| 3134 |  |  |  |  |  |  | -3670.37 | -1.169 |
| 3135 |  |  |  |  |  |  | -3890.32 | -1.1223 |
| 3136 |  |  |  |  |  |  | -3791.41 | -1.084 |
| 3137 |  |  |  |  |  |  | -3827.93 | -1.077 |
| 3138 |  |  |  |  |  |  | -3853.15 | -1.085 |
| 3139 |  |  |  |  |  | 23.533 | -3534.06 | -1.1747 |
| 3140 |  |  | 12454.16667 | 0 |  | 0 |  |  |
| 3291 |  |  |  |  |  | 0.601 | -284.41 | -1.0261 |
| 3292 |  |  |  |  |  |  | -13.46 | -1.0713 |
| 3299 |  |  |  |  |  |  | -42.83 | -1.636 |
| 3300 |  |  |  |  |  |  | -250.10 | -1.6092 |
| 3301 |  |  |  |  |  |  | -541.76 | -1.5995 |
| 3302 |  |  |  |  |  |  | -933.34 | -1.6353 |
| 3303 |  |  |  |  |  |  | -1699.27 | -1.6595 |
| 3304 |  |  |  |  |  |  | -2173.44 | -1.6608 |
| 3305 |  |  |  |  |  |  | -2333.99 | -1.6607 |
| 3306 |  |  |  |  |  |  | -2641.36 | -1.6605 |
| 3307 |  |  |  |  |  |  | -2937.50 | -1.6595 |
| 3308 |  |  |  |  |  |  | -3335.94 | -1.6643 |
| 3309 |  |  |  |  |  |  | -3727.33 | -1.6673 |
| 3310 |  |  |  |  |  |  | -3887.68 | -1.5913 |
| 3311 |  |  |  |  |  |  | -3891.79 | -1.4703 |
| 3312 |  |  |  |  |  |  | -3891.63 | -1.3495 |
| 3313 |  |  |  |  |  |  | -4083.67 | -1.2027 |
| 3314 |  |  |  |  |  |  | -4152.71 | -1.1085 |
| 3315 |  |  |  |  |  |  | -4110.92 | -1.0249 |
| 3316 |  |  |  |  |  |  | -4097.14 | -0.9248 |
| 3317 |  |  |  |  |  |  | -4117.43 | -0.9027 |
| 3318 |  |  |  |  |  |  | -4241.99 | -0.9277 |
| 3319 |  |  |  |  |  |  | -4314.13 | -0.9255 |
| 3471 |  |  |  |  |  | 23.4345 | -411.69 | -1.3143 |
| 3472 |  |  |  |  |  |  | -15.22 | -1.434 |
| 3479 |  |  |  |  |  |  | -159.58 | -1.622 |
| 3480 |  |  |  |  |  |  | -397.15 | -1.6253 |
| 3481 |  |  |  |  |  |  | -652.28 | -1.6653 |
| 3482 |  |  |  |  |  |  | -1604.25 | -1.6865 |
| 3483 |  |  |  |  |  |  | -2730.03 | -1.685 |
| 3484 |  |  |  |  |  |  | -2937.91 | -1.681 |
| 3485 |  |  |  |  |  |  | -3229.72 | -1.647 |
| 3486 |  |  |  |  |  |  | -3487.34 | -1.6403 |
| 3487 |  |  |  |  |  |  | -3582.97 | -1.6203 |
| 3488 |  |  |  |  |  |  | -3666.81 | -1.589 |
| 3489 |  |  |  |  |  |  | -3949.77 | -1.5303 |
| 3490 |  |  |  |  |  |  | -4151.38 | -1.4383 |
| 3491 |  |  |  |  |  |  | -4170.92 | -1.306 |
| 3492 |  |  |  |  |  |  | -4195.75 | -1.135 |
| 3493 |  |  |  |  |  |  | -4295.24 | -1.0123 |
| 3494 |  |  |  |  |  |  | -4385.17 | -0.9019 |
| 3495 |  |  |  |  |  |  | -4450.22 | -0.84 |
| 3496 |  |  |  |  |  |  | -4443.54 | -0.7506 |
| 3497 |  |  |  |  |  |  | -4381.14 | -0.7102 |
| 3498 |  |  |  |  |  |  | -4424.54 | -0.7019 |
| 3499 |  |  |  |  |  |  | -4476.48 | -0.6324 |
| 3651 |  |  |  |  |  | 71.24133333 | -449.15 | -0.9177 |
| 3652 |  |  |  |  |  | 88.47265217 |  |  |
| 3653 |  |  | 83.59 | 874.2 |  |  |  |  |
| 3655 |  |  | 58.50916667 | 783.1033333 |  |  |  |  |
| 3659 |  |  |  |  |  |  | -413.01 | -1.616 |
| 3660 |  |  |  |  |  |  | -457.89 | -1.6207 |
| 3661 |  |  |  |  |  |  | -587.03 | -1.6657 |
| 3662 |  |  |  |  |  |  | -1763.56 | -1.69 |
| 3663 |  |  |  |  |  |  | -2941.17 | -1.69 |
| 3664 |  |  |  |  |  |  | -3267.05 | -1.688 |
| 3665 |  |  |  |  |  |  | -3524.16 | -1.6655 |
| 3666 |  |  |  |  |  |  | -3814.90 | -1.6177 |
| 3667 |  |  |  |  |  |  | -3950.48 | -1.5523 |
| 3668 |  |  |  |  |  |  | -3918.12 | -1.5025 |
| 3669 |  |  |  |  |  |  | -4000.14 | -1.4433 |
| 3670 |  |  |  |  |  |  | -4242.56 | -1.307 |
| 3671 |  |  |  |  |  |  | -4286.38 | -1.177 |
| 3672 |  |  |  |  |  |  | -4354.21 | -1.0054 |
| 3673 |  |  |  |  |  |  | -4432.56 | -0.8814 |
| 3674 |  |  |  |  |  |  | -4497.22 | -0.7875 |
| 3675 |  |  |  |  |  |  | -4582.97 | -0.7408 |
| 3676 |  |  |  |  |  |  | -4646.12 | -0.6685 |
| 3677 |  |  |  |  |  |  | -4623.75 | -0.6026 |
| 3678 |  |  |  |  |  |  | -4633.53 | -0.543 |
| 3679 |  |  |  |  |  |  | -4663.49 | -0.4708 |
| 3830 |  |  |  |  |  | 1.714 |  |  |
| 3831 |  |  |  |  |  | 11.61933333 | -2467.88 | -0.0381 |
| 3832 |  |  |  |  |  | 74.44322222 | -769.65 | -0.5313 |
| 3833 |  |  |  |  |  | 51.2709 | -373.60 | -0.8091 |
| 3834 |  | 0 | 90.50126663 | 316.51975 |  | 9.736363636 | -369.26 | -1.039 |
| 3835 |  | 0 | 57.46089059 | 74.01740752 |  | 26.66846154 | -459.76 | -1.3304 |
| 3836 |  | 0 | 0.155 | 384.9175 |  | 7.088 | -179.87 | -1.4645 |
| 3840 |  |  |  |  |  |  | -473.64 | -1.6258 |
| 3841 |  |  |  |  |  |  | -467.12 | -1.6783 |
| 3842 |  |  |  |  |  |  | -1779.61 | -1.689 |
| 3843 |  |  |  |  |  |  | -2862.63 | -1.6832 |
| 3844 |  |  |  |  |  |  | -3181.06 | -1.6748 |
| 3845 |  |  |  |  |  |  | -3415.80 | -1.6278 |
| 3846 |  |  |  |  |  |  | -3570.31 | -1.5293 |
| 3847 |  |  |  |  |  |  | -3909.15 | -1.4198 |
| 3848 |  |  |  |  |  |  | -4167.54 | -1.3068 |
| 3849 |  |  |  |  |  |  | -4216.83 | -1.2063 |
| 3850 |  |  |  |  |  |  | -4304.07 | -1.0245 |
| 3851 |  |  |  |  |  |  | -4390.33 | -0.8599 |
| 3852 |  |  |  |  |  |  | -4448.60 | -0.7293 |
| 3853 |  |  |  |  |  |  | -4499.56 | -0.652 |
| 3854 |  |  |  |  |  |  | -4578.38 | -0.6061 |
| 3855 |  |  |  |  |  |  | -4645.81 | -0.5788 |
| 3856 |  |  |  |  |  |  | -4733.10 | -0.5162 |
| 3857 |  |  |  |  |  |  | -4800.34 | -0.4345 |
| 3858 |  |  |  |  |  |  | -4835.45 | -0.3502 |
| 3859 |  |  |  |  |  |  | -4860.43 | -0.2577 |
| 4011 |  |  |  |  |  | 42.5595 | -3519.20 | 0.9595 |
| 4012 |  | 0 | 187.5732708 | 0 |  | 30.314 | -2412.81 | 0.6698 |
| 4013 |  | 0 | 3290.720094 | 0 |  | 83.52933333 | -851.74 | 0.4779 |
| 4014 |  | 0 | 42.03376342 | 5.796148027 |  | 39.58091667 | -535.46 | 0.2642 |
| 4015 |  | 0 | 4.609143113 | 115.441075 |  | 66.28462921 | -273.62 | -0.808 |
| 4016 |  | 0 | 0 | 104.2258333 |  | 20.3738 |  |  |
| 4019 |  |  |  |  |  |  | -295.33 | -1.632 |
| 4020 |  |  |  |  |  |  | -398.24 | -1.647 |
| 4021 |  |  |  |  |  |  | -526.85 | -1.6713 |
| 4022 |  |  |  |  |  |  | -1801.73 | -1.6715 |
| 4023 |  |  |  |  |  |  | -3006.24 | -1.622 |
| 4024 |  |  |  |  |  |  | -3429.44 | -1.5957 |
| 4025 |  |  |  |  |  |  | -3662.49 | -1.5195 |
| 4026 |  |  |  |  |  |  | -3850.20 | -1.3703 |
| 4027 |  |  |  |  |  |  | -4015.84 | -1.1987 |
| 4028 |  |  |  |  |  |  | -4211.46 | -1.0228 |
| 4029 |  |  |  |  |  |  | -4369.60 | -0.8823 |
| 4030 |  |  |  |  |  |  | -4424.52 | -0.6482 |
| 4031 |  |  |  |  |  |  | -4492.23 | -0.5255 |
| 4032 |  |  |  |  |  |  | -4553.72 | -0.4241 |
| 4033 |  |  |  |  |  |  | -4603.81 | -0.3543 |
| 4034 |  |  |  |  |  |  | -4655.40 | -0.3288 |
| 4035 |  |  |  |  |  |  | -4723.87 | -0.359 |
| 4036 |  |  |  |  |  |  | -4790.97 | -0.349 |
| 4037 |  |  |  |  |  |  | -4853.68 | -0.2831 |
| 4038 |  |  |  |  |  |  | -4896.12 | -0.2047 |
| 4039 |  |  |  |  |  |  | -4917.37 | -0.0928 |
| 4191 |  |  |  |  |  | 0 | -3730.86 | 1.3087 |
| 4192 |  | 0 | 116.7262076 | 0 |  | 6.295 | -3532.33 | 1.1867 |
| 4193 |  | 0 | 2634.492344 | 7430 |  | 4.480693333 | -2904.33 | 1.1015 |
| 4194 |  | 0 | 6391.39335 | 5235.150537 |  | 9.100480519 | -1012.48 | 0.9459 |
| 4195 | 6.588181818 | 3.102857143 | 2.477241879 | 456.977486 | 7.3155 | 84.29249462 | -464.25 | -0.0545 |
| 4196 | 0 | 0 | 333.4975 | 13.338125 |  | 40.15870787 | -237.74 | -1.028 |
| 4197 |  |  |  |  |  | 6.5945 |  |  |
| 4200 |  |  |  |  |  |  | -439.87 | -1.643 |
| 4201 |  |  |  |  |  |  | -539.73 | -1.658 |
| 4202 |  |  |  |  |  |  | -1324.34 | -1.6425 |
| 4203 |  |  |  |  |  |  | -2766.15 | -1.5817 |
| 4204 |  |  |  |  |  |  | -3249.42 | -1.5163 |
| 4205 |  |  |  |  |  | 70.276 | -3739.11 | -1.412 |
| 4206 |  |  |  |  |  |  | -4084.16 | -1.2353 |
| 4207 | 0 | 0 | 0 | 0 |  | 0.241 | -4268.46 | -1.0438 |
| 4208 | 0 | 0 | 0 | 0 |  | 0.098 | -4344.43 | -0.8815 |
| 4209 |  |  |  |  |  |  | -4473.26 | -0.7048 |
| 4210 |  |  |  |  |  |  | -4532.28 | -0.5015 |
| 4211 |  |  |  |  |  |  | -4607.21 | -0.4097 |
| 4212 |  |  |  |  |  |  | -4696.98 | -0.3281 |
| 4213 |  |  |  |  |  |  | -4751.21 | -0.2717 |
| 4214 |  |  |  |  |  |  | -4774.26 | -0.2413 |
| 4215 |  |  |  |  |  |  | -4824.49 | -0.2481 |
| 4216 |  |  |  |  |  |  | -4852.04 | -0.243 |
| 4217 |  |  |  |  |  |  | -4892.38 | -0.2027 |
| 4218 |  |  |  |  |  |  | -4925.97 | -0.1231 |
| 4219 |  |  |  |  |  |  | -4934.90 | -0.0303 |
| 4371 |  |  |  |  |  |  | -3997.77 | 1.7455 |
| 4372 |  |  |  |  |  |  | -3904.12 | 1.7338 |
| 4373 | 0 | 0 | 4146.625947 | 25.09410289 |  | 0.322 | -3536.43 | 1.6763 |
| 4374 | 0 | 0 | 30438.08466 | 30.73229292 | 0.106 | 3.856829268 | -2988.22 | 1.5933 |
| 4375 | 0 | 0 | 3668.916694 | 1.893402778 | 0.7375 | 20.76856522 | -1020.79 | 1.3007 |
| 4376 | 1.48 | 0 | 3.08851995 | 0.252661974 | 1.146 | 51.07611957 | -378.40 | 0.3868 |
| 4377 | 0 | 0 | 2.367223065 | 0 |  | 56.12153846 | -157.50 | -0.1298 |
| 4378 |  |  |  |  |  | 159.394 |  |  |
| 4380 |  |  |  |  |  |  | -351.50 | -1.6373 |
| 4381 |  |  |  |  |  |  | -426.39 | -1.5945 |
| 4382 |  |  |  |  |  |  | -614.84 | -1.5498 |
| 4383 |  |  |  |  |  |  | -2162.69 | -1.5038 |
| 4384 |  |  |  |  |  | 14.236 | -3094.84 | -1.3672 |
| 4385 |  |  |  |  |  | 79.261 | -3758.64 | -1.164 |
| 4386 |  |  |  |  |  | 46.233 | -4145.30 | -0.9831 |
| 4387 | 0 | 0 | 6 | 0 |  |  | -4351.15 | -0.7867 |
| 4388 | 0 | 0 | 0 | 0 |  | 0.091 | -4501.18 | -0.6004 |
| 4389 |  |  |  |  |  |  | -4613.55 | -0.4835 |
| 4390 |  |  |  |  |  |  | -4675.09 | -0.3944 |
| 4391 |  |  |  |  |  |  | -4711.70 | -0.3391 |
| 4392 |  |  |  |  |  |  | -4785.06 | -0.2744 |
| 4393 |  |  |  |  |  |  | -4805.66 | -0.2266 |
| 4394 |  |  |  |  |  |  | -4835.90 | -0.1976 |
| 4395 |  |  |  |  |  |  | -4881.31 | -0.1877 |
| 4396 |  |  |  |  |  |  | -4893.65 | -0.1673 |
| 4397 |  |  |  |  |  |  | -4908.21 | -0.1326 |
| 4398 |  |  |  |  |  |  | -4967.85 | -0.0736 |
| 4399 |  |  |  |  |  |  | -4987.27 | 0.0064 |
| 4551 |  |  |  |  |  |  | -4152.82 | 2.3417 |
| 4552 |  |  |  |  |  |  | -3760.11 | 2.297 |
| 4553 |  |  |  |  |  |  | -3741.63 | 2.181 |
| 4554 | 0 | 0 | 66.36447264 | 2.530644524 |  | 0.214 | -3489.09 | 2.0277 |
| 4555 | 0 | 0 | 1142.952616 | 0 | 1.832 | 3.71545098 | -2720.81 | 1.8967 |
| 4556 | 0 | 0 | 28.65369967 | 0.343200343 | 0.137 | 4.90697619 | -1015.82 | 1.5955 |
| 4557 | 4.439 | 0.860833333 | 22.09611201 | 0.401488834 | 52.93542857 | 26.96288649 | -438.34 | 1.0339 |
| 4558 | 1.947 | 0 | 7.666392761 | 0.312778955 | 126.0156923 | 163.6853043 | -241.47 | -0.6147 |
| 4559 | 0 | 0 | 1535.896522 | 287.42 | 0 | 103.5272273 |  |  |
| 4561 |  |  |  |  |  | 0.189 | -282.92 | -1.4545 |
| 4562 |  |  |  |  |  | 28.38066667 | -478.47 | -1.264 |
| 4563 |  |  |  |  |  | 0.4675 | -2190.48 | -1.224 |
| 4564 |  |  |  |  |  | 210.592 | -2978.58 | -1.0704 |
| 4565 |  |  |  |  |  |  | -3717.70 | -0.8098 |
| 4566 | 0 | 0 | 0 | 0 |  |  | -4228.43 | -0.6954 |
| 4567 | 0 | 0 | 12 | 0 |  | 5.733 | -4451.52 | -0.5077 |
| 4568 | 0 | 0 | 0.2 | 0 |  | 42.548 | -4593.58 | -0.3564 |
| 4569 |  |  |  |  |  |  | -4692.16 | -0.352 |
| 4570 |  |  |  |  |  |  | -4776.78 | -0.3546 |
| 4571 |  |  |  |  |  |  | -4766.67 | -0.3148 |
| 4572 |  |  |  |  |  |  | -4741.29 | -0.262 |
| 4573 |  |  |  |  |  |  | -4788.56 | -0.2206 |
| 4574 |  |  |  |  |  |  | -4843.02 | -0.2014 |
| 4575 |  |  |  |  |  |  | -4874.72 | -0.1795 |
| 4576 |  |  |  |  |  |  | -4875.59 | -0.131 |
| 4577 |  |  |  |  |  |  | -4939.07 | -0.0862 |
| 4578 |  |  |  |  |  |  | -4975.10 | -0.0365 |
| 4579 |  |  |  |  |  |  | -4994.24 | 0.0335 |
| 4731 |  |  |  |  |  |  | -4053.85 | 2.847 |
| 4732 |  |  |  |  |  |  | -4051.59 | 2.7507 |
| 4733 |  |  |  |  |  |  | -3955.46 | 2.5885 |
| 4734 |  |  |  |  |  |  | -3810.71 | 2.4227 |
| 4735 | 0 | 0 | 411.4461019 | 0 | 0.119 | 1.97 | -3535.33 | 2.1823 |
| 4736 | 0 | 0 | 63.64675389 | 1.127954495 | 0.0445 | 2.01962963 | -3273.43 | 1.963 |
| 4737 | 0 | 0 | 463.3268384 | 0.13125 | 11.2566 | 7.149785714 | -1538.17 | 1.5957 |
| 4738 | 1.88 | 0 | 2.975555556 | 0.217849693 | 8.542692308 | 4.302166667 | -369.62 | 0.5519 |
| 4739 | 583.1918182 | 8969.046154 | 896.4287731 | 50.41808034 | 115.5534412 | 35.00638298 | -539.89 | 0.6811 |
| 4740 | 1.1405 | 2.586666667 | 96.38906573 | 19.26819186 | 7.033 | 140.1715476 | -336.80 | -1.0179 |
| 4741 | 1854.532143 | 3804.975714 | 485.4505 | 0.542 | 9.4645 | 25.155375 | -281.70 | -1.3843 |
| 4742 | 0 | 0 | 191.6666667 | 0 | 0.01725 | 35.03653846 | -268.49 | -0.9753 |
| 4743 | 3.5 | 0 | 183.344 | 0 |  | 128.45625 | -649.07 | -0.9044 |
| 4744 | 0 | 0 | 1 | 0 |  | 3.332 | -1969.67 | -0.7506 |
| 4745 | 1.808333333 | 0.466666667 | 5.6225 | 0.107 | 0.048 | 8.227 | -3065.63 | -0.6199 |
| 4746 | 0 | 0 | 3 | 0 |  |  | -3403.54 | -0.4726 |
| 4747 | 0 | 0 | 0.2 | 0 |  | 0.881 | -3740.88 | -0.3381 |
| 4748 | 0 | 0 | 0.2 | 0 |  | 18.165 | -3944.86 | -0.2727 |
| 4749 | 0 | 0 | 13.6 | 12.2 |  |  | -4320.90 | -0.2976 |
| 4750 | 0 | 0 | 1 | 0 |  |  | -4599.94 | -0.3369 |
| 4751 |  |  |  |  |  |  | -4663.08 | -0.3081 |
| 4752 | 0 | 0 | 4.8 | 0 |  |  | -4686.92 | -0.2448 |
| 4753 | 0 | 0 | 0 | 0 |  |  | -4671.30 | -0.2031 |
| 4754 | 0 | 0 | 0 | 0 |  |  | -4765.85 | -0.1861 |
| 4755 | 0 | 0 | 0 | 0 |  |  | -4769.46 | -0.1614 |
| 4756 |  |  |  |  |  |  | -4813.42 | -0.1096 |
| 4757 |  |  |  |  |  |  | -4846.81 | -0.0368 |
| 4758 |  |  |  |  |  |  | -4952.82 | 0.0173 |
| 4759 | 0 | 0 | 0 | 0 |  |  | -4918.98 | 0.0731 |
| 4911 |  |  |  |  |  |  | -4636.81 | 3.9657 |
| 4912 |  |  |  |  |  |  | -4352.91 | 3.7972 |
| 4913 |  |  |  |  |  |  | -4219.26 | 3.503 |
| 4914 |  |  |  |  |  |  | -4080.66 | 3.243 |
| 4915 |  |  |  |  |  |  | -3938.39 | 2.8455 |
| 4916 | 0 | 0 | 50.45814905 | 0.509138381 |  | 11.98214286 | -3553.35 | 2.4475 |
| 4917 | 0 | 0 | 547.2415183 | 0 | 0.0665 | 7.994333333 | -3382.18 | 2.1668 |
| 4918 | 0 | 0 | 707.3812193 | 3.887289306 | 0.606222222 | 4.993413793 | -2610.27 | 1.7978 |
| 4919 | 10.8068 | 0 | 726.8509566 | 9.833183622 | 15.22023077 | 14.03728859 | -527.91 | 1.203 |
| 4920 | 1.528833333 | 1.008 | 825.6028636 | 65.54711512 | 208.0743103 | 27.14357838 | -782.53 | 0.5178 |
| 4921 | 16.63444444 | 11.74666667 | 672.7584129 | 100.4635424 | 32.29324242 | 52.68710487 | -786.15 | 0.0714 |
| 4922 | 54.75 | 0 | 3.746989887 | 0.256597265 | 71.38407407 | 128.9523973 | -410.92 | 0.0471 |
| 4923 | 1971 | 0 | 3.777777778 | 0.111111111 | 10.83 | 52.89988889 | -1864.56 | -0.1467 |
| 4924 | 9 | 0 | 13.47288889 | 0 |  | 5.5636 | -3222.89 | -0.2091 |
| 4925 | 17.175 | 0 | 69.61416667 | 0 | 44.6975 | 4.823 | -3276.05 | -0.1656 |
| 4926 |  |  |  |  | 66.5676 |  | -2977.67 | -0.0193 |
| 4927 | 0 | 0 | 4181.76 | 0 |  | 1125.757333 | -2083.38 | 0.0488 |
| 4928 | 0 | 0 | 0.6 | 0 |  |  | -2344.14 | 0.0217 |
| 4929 |  |  |  |  |  |  | -3352.34 | -0.0896 |
| 4930 |  |  |  |  | 4.785 |  | -3658.31 | -0.1812 |
| 4931 |  |  |  |  | 1.271 |  | -4360.55 | -0.1781 |
| 4932 |  |  |  |  | 3.07 |  | -4374.43 | -0.0937 |
| 4933 |  |  |  |  |  | 104.9605 | -4377.34 | -0.0689 |
| 4934 |  |  |  |  |  |  | -4428.44 | -0.0779 |
| 4935 | 0 | 0 | 0 | 0 |  | 0 | -4426.25 | -0.0672 |
| 4936 |  |  |  |  |  |  | -4658.57 | 0.0112 |
| 4937 |  |  |  |  |  |  | -4677.31 | 0.1079 |
| 4938 |  |  |  |  |  |  | -4700.47 | 0.1783 |
| 4939 |  |  |  |  |  |  | -4796.43 | 0.2187 |
| 5091 |  |  |  |  |  |  | -4698.08 | 4.9377 |
| 5092 |  |  |  |  |  |  | -4517.68 | 4.7903 |
| 5093 |  |  |  |  |  |  | -4402.14 | 4.4815 |
| 5094 |  |  |  |  |  |  | -4220.72 | 4.1567 |
| 5095 | 0 | 0 | 0 | 0 |  | 10.621 | -4052.63 | 3.693 |
| 5096 | 0 | 0 | 0 | 0 |  | 90.7085 | -3824.26 | 3.234 |
| 5097 | 0 | 0 | 420.8483563 | 0 |  | 0 | -3416.02 | 2.8513 |
| 5098 | 3.172 | 68.7 | 973.5813429 | 1.329115933 | 0.113666667 | 13.72431429 | -3626.55 | 2.444 |
| 5099 | 0 | 0 | 2105.75848 | 51.43414458 | 25.031 | 6.052147651 | -3685.68 | 2.1395 |
| 5100 | 0 | 0 | 1201.320383 | 39.5 | 51.279125 | 26.47819289 | -2276.00 | 1.8217 |
| 5101 | 12.73125 | 5.240833333 | 693.5926726 | 18.84254854 | 94.71792537 | 13.99296517 | -1087.06 | 1.503 |
| 5102 | 5.131882353 | 35.23027778 | 6583.517156 | 78.8276323 | 43.10255263 | 96.21403822 | -795.98 | 1.0371 |
| 5103 | 0 | 0 | 137.4842667 | 6.066666667 | 183.7025882 | 11.7666879 | -1006.96 | 0.6103 |
| 5104 | 0 | 0 | 712.6714286 | 0 | 2.447 | 35.784 | -2287.42 | 0.3607 |
| 5105 | 1.085333333 | 0 | 0.6456 | 0 | 0.6315 | 0.949714286 | -3035.77 | 0.3601 |
| 5106 | 0 | 0 | 81.928 | 15.072 | 392.12 | 6.109222222 | -1308.46 | 0.4874 |
| 5107 | 0 | 109.5 | 0 | 0 | 2.404 | 164.6623571 | -366.26 | 0.5462 |
| 5108 | 7.216 | 0 | 2.605333333 | 0 |  | 323.3748 | -626.90 | 0.5013 |
| 5109 |  |  |  |  |  | 0.485 | -2410.64 | 0.3541 |
| 5110 | 0 | 0 | 3.8 | 48 | 4.924333333 |  | -3077.66 | 0.2319 |
| 5111 | 0 | 0 | 3.289 | 0 | 1337.212286 | 24.326 | -2678.38 | 0.2183 |
| 5112 | 0 | 0 | 0 | 0 |  |  | -3181.50 | 0.2236 |
| 5113 |  |  |  |  |  |  | -3251.29 | 0.1847 |
| 5114 | 0 | 0 | 0 | 0 |  | 25.172 | -3751.01 | 0.1359 |
| 5115 |  |  |  |  |  |  | -4016.56 | 0.1278 |
| 5116 |  |  |  |  |  |  | -4107.65 | 0.2213 |
| 5117 |  |  |  |  |  |  | -4212.39 | 0.3428 |
| 5118 |  |  |  |  |  | 516.8201 | -4441.83 | 0.4404 |
| 5119 |  |  |  |  |  | 429.057 | -4326.44 | 0.4773 |
| 5271 |  |  |  |  |  |  | -4906.07 | 5.4503 |
| 5272 |  |  |  |  |  |  | -4519.79 | 5.35 |
| 5273 |  |  |  |  |  |  | -4274.52 | 5.1185 |
| 5274 |  |  |  |  |  |  | -4236.23 | 4.746 |
| 5275 |  |  |  |  |  |  | -4018.57 | 4.34 |
| 5276 |  |  |  |  |  |  | -3325.16 | 3.8545 |
| 5277 | 0 | 0 | 0 | 2.502360718 |  | 2.431333333 | -3247.80 | 3.387 |
| 5278 | 0 | 0 | 18000 | 0 | 0.009 | 0 | -3739.61 | 2.9447 |
| 5279 | 0 | 0 | 107.5 | 0 | 0 | 3.443461538 | -3825.26 | 2.628 |
| 5280 | 0 | 0 | 1691.8 | 780.5523908 | 84.16963636 | 1.046807018 | -3984.58 | 2.393 |
| 5281 | 1.036 | 0 | 988.3669151 | 29.5608793 | 23.30708163 | 8.653979592 | -3571.10 | 2.0453 |
| 5282 | 13.08663158 | 106.7919536 | 1316.860048 | 53.03185452 | 91.06243529 | 24.12390018 | -2796.61 | 1.621 |
| 5283 | 0 | 0 | 15.768 | 0.2 | 28.21552 | 18.66568205 | -1989.16 | 1.1913 |
| 5284 | 5.225833333 | 60.44583333 | 78.17875 | 0 | 0.936 | 7.061 | -2329.82 | 0.7925 |
| 5285 | 55.42375 | 0 | 74680.19811 | 27.02551167 | 13.83266667 | 12.45095238 | -2303.41 | 0.7269 |
| 5286 | 13.11466667 | 54.75 | 6195.9675 | 1.752166667 | 39.16675 | 70.89128947 | -1528.80 | 0.8669 |
| 5287 | 124.556 | 0 | 1065.438352 | 65.58110064 | 14.28518182 | 19.80742553 | -2068.59 | 0.8967 |
| 5288 | 175.2 | 13.6875 | 14453.92771 | 312.1002857 | 67.66933333 | 21.145125 | -2802.77 | 0.9079 |
| 5289 | 0 | 0 | 125.674 | 0 | 15.87 | 3.233857143 | -2855.56 | 0.8009 |
| 5290 | 0 | 0 | 221.868 | 0 | 16.0989 | 1.654 | -2705.50 | 0.6645 |
| 5291 |  |  |  |  | 9.247 |  | -1832.24 | 0.6101 |
| 5292 | 0 | 0 | 0 | 0 |  | 50.073 | -2473.92 | 0.5521 |
| 5293 | 0 | 0 | 0 | 0 |  | 0 | -2381.19 | 0.4432 |
| 5294 | 0 | 0 | 0 | 0 |  | 15.74733333 | -2327.95 | 0.3583 |
| 5295 | 0 | 0 | 27.5 | 0 |  | 0.49 | -2646.44 | 0.3049 |
| 5296 | 0 | 0 | 0 | 0 |  | 0 | -2791.15 | 0.4389 |
| 5297 | 0 | 0 | 0 | 0 |  | 0 | -4707.06 | 0.6381 |
| 5298 |  |  |  |  |  |  | -4058.24 | 0.7606 |
| 5299 |  |  |  |  |  |  | -3962.45 | 0.7502 |
| 5453 |  |  |  |  |  |  | -4436.75 | 5.9653 |
| 5454 |  |  |  |  |  |  | -4099.74 | 5.6892 |
| 5455 |  |  |  |  |  |  | -3623.22 | 5.3218 |
| 5456 |  |  |  |  |  |  | -3567.85 | 4.9355 |
| 5457 |  |  |  |  |  |  | -3375.33 | 4.385 |
| 5458 |  |  |  |  |  |  | -3901.24 | 3.7525 |
| 5459 |  |  |  |  |  | 0 | -3861.92 | 3.4627 |
| 5460 | 0 | 0 | 0 | 0 |  | 1.1716 | -3513.94 | 3.1492 |
| 5461 | 3.5 | 116 | 20 | 0 | 3.2554 | 23.78089855 | -3609.49 | 2.807 |
| 5462 | 0 | 0 | 37795.5 | 22 | 16.59033333 | 6.300079545 | -3674.51 | 2.5303 |
| 5463 | 0 | 0 | 37236.83333 | 4838.5 | 275.3296 | 11.73217778 | -3525.57 | 2.183 |
| 5464 | 0.055 | 0 | 33155.53556 | 0 | 25.02866667 | 12.05154545 | -3197.69 | 1.7485 |
| 5465 | 0 | 0 | 4388.71 | 53.75 | 19.133 | 29.27642857 | -3984.61 | 1.61 |
| 5466 | 219 | 0 | 17775.36017 | 1651.254833 | 130.0995714 | 3.154733333 | -3714.85 | 1.6337 |
| 5467 | 0 | 7.821428571 | 5610.809031 | 387.94626 | 37.56785714 | 6.45565625 | -2823.93 | 1.645 |
| 5468 | 0 | 0 | 6726.536 | 481.345 | 230.6993333 | 22.35383333 | -3905.20 | 1.6107 |
| 5469 | 0 | 0 | 0 | 0 | 38.142875 | 4.741 | -3069.14 | 1.501 |
| 5470 | 0 | 0 | 10991.456 | 58.882 | 9.015 | 24.006 | -2436.87 | 1.3178 |
| 5471 | 0 | 0 | 11046.444 | 29.876 | 4.96525 | 0.8415 | -2626.48 | 1.1837 |
| 5472 | 0 | 0 | 0 | 0 |  | 606.7456667 | -2041.47 | 1.038 |
| 5473 | 0 | 0 | 41.065 | 0 |  | 0.066 | -2633.45 | 0.8648 |
| 5474 |  |  |  |  |  | 243.5225 | -2815.39 | 0.7024 |
| 5475 | 0 | 0 | 0 | 0 |  | 2073.191 | -2639.93 | 0.621 |
| 5476 | 0 | 0 | 0 | 0 |  | 64.28233333 | -1783.17 | 0.7013 |
| 5477 | 0 | 0 | 0 | 0 |  | 0 | -3970.83 | 0.9119 |
| 5478 | 0 | 0 | 0.228 | 0 |  | 0 | -4529.40 | 1.0954 |
| 5479 | 0 | 0 | 0 | 0 |  | 313.787 | -4145.06 | 1.0741 |
| 5637 |  |  |  |  |  |  | -3333.31 | 5.501 |
| 5638 |  |  |  |  |  |  | -3640.92 | 4.8627 |
| 5639 |  |  |  |  |  |  | -3341.51 | 4.3605 |
| 5640 |  |  |  |  |  |  | -3618.64 | 3.93 |
| 5641 |  |  |  |  |  |  | -3856.58 | 3.615 |
| 5642 | 0 | 191.63 | 0 | 0 |  | 0 | -3999.58 | 3.475 |
| 5643 |  |  |  |  |  |  | -3893.74 | 3.2173 |
| 5644 | 0 | 0 | 70981.80286 | 4893.857143 | 0.57 | 1.149 | -3546.71 | 2.7497 |
| 5645 | 0 | 273.75 | 44226.40514 | 43.85714286 | 25.2515 | 160.7208333 | -3611.60 | 2.54 |
| 5646 | 8.182 | 0 | 1929.379 | 347.441 | 52.9502 | 17.08475 | -3041.34 | 2.4407 |
| 5647 | 0 | 0 | 1580.649444 | 289.0538889 | 29.0185 | 5.489875 | -2628.67 | 2.3313 |
| 5648 | 0 | 0 | 4642.745167 | 783.0270182 |  | 39.38969231 | -2920.78 | 2.2855 |
| 5649 | 0 | 0 | 1702.339 | 0 |  | 3.409666667 | -3146.75 | 2.2133 |
| 5650 |  |  |  |  | 1.8602 | 0 | -3001.14 | 2.0587 |
| 5651 | 0 | 0 | 329 | 0 | 0.4895 | 0.858 | -2626.47 | 1.8315 |
| 5652 | 0 | 0 | 620.6666667 | 0 |  | 4.293333333 | -2615.04 | 1.6367 |
| 5653 | 0 | 0 | 0 | 0 |  | 0.393 | -3246.49 | 1.4473 |
| 5654 |  |  |  |  |  | 2.44 | -3374.45 | 1.187 |
| 5655 | 73 | 0 | 45.5 | 0 |  | 0 | -3194.70 | 0.9742 |
| 5656 | 0 | 0 | 0.472 | 0 |  | 4.8592 | -2309.95 | 0.9721 |
| 5657 | 0 | 0 | 0 | 0 |  | 57.343 | -2981.60 | 1.144 |
| 5658 |  |  |  |  |  |  | -5179.72 | 1.4063 |
| 5659 |  |  |  |  |  |  | -4328.09 | 1.406 |
| 5818 |  |  |  |  |  |  | -3850.87 | 5.6773 |
| 5819 |  |  |  |  |  |  | -3695.10 | 5.217 |
| 5820 |  |  |  |  |  |  | -3454.56 | 4.772 |
| 5821 |  |  |  |  |  |  | -3783.45 | 4.3027 |
| 5822 | 0 | 0 | 11 | 5.4 | 0 | 0 | -4081.10 | 4.151 |
| 5823 |  |  |  |  |  |  | -4231.03 | 3.964 |
| 5824 | 0 | 0 | 125.27364 | 0 | 3.161166667 | 1.409571429 | -3916.24 | 3.5363 |
| 5825 | 0 | 0 | 3715.2752 | 26.6628 |  | 2.982666667 | -3789.44 | 3.267 |
| 5826 |  |  |  |  | 2.555 | 1.062 | -3223.45 | 3.0113 |
| 5827 | 0 | 109.5 | 3530.1126 | 650.5138 | 418.8 | 0.089333333 | -3114.69 | 2.8097 |
| 5828 | 0 | 0 | 18138.32541 | 1594.754 | 147.661 | 2.118 | -3120.89 | 2.6995 |
| 5829 | 0 | 0 | 3046.332 | 87.39333333 | 0.775 | 0.766 | -3434.17 | 2.6473 |
| 5830 | 0 | 0 | 122.8 | 0 | 7918.263 |  | -2935.05 | 2.508 |
| 5831 | 4.744 | 0 | 3223.974667 | 1846.725333 |  | 20.1508 | -3155.31 | 2.2835 |
| 5832 | 0 | 0 | 0 | 0 | 0 | 126.5803333 | -3338.58 | 2.066 |
| 5833 |  |  |  |  |  |  | -3342.95 | 1.8103 |
| 5834 | 0 | 0 | 109.5 | 0 | 1.217 | 9.134 | -3495.65 | 1.497 |
| 5835 | 0 | 0 | 0.542 | 0 |  | 0 | -3321.54 | 1.199 |
| 5836 | 0 | 0 | 0 | 0 |  | 63.662 | -2435.17 | 1.156 |
| 5837 |  |  |  |  |  |  | -4248.05 | 1.3445 |
| 5838 | 0 | 0 | 0 | 0 |  | 2.221 | -5096.29 | 1.6187 |
| 5839 | 0 | 0 | 0 | 0 |  |  | -4478.32 | 1.6243 |
| 6001 |  |  |  |  |  |  | -3604.97 | 5.5912 |
| 6002 |  |  |  |  |  |  | -4019.31 | 5.2995 |
| 6003 |  |  |  |  |  |  | -4023.13 | 5.0705 |
| 6004 | 0 | 0 | 0 | 0 |  | 0 | -4212.49 | 4.7323 |
| 6005 |  |  |  |  |  | 0 | -3852.12 | 4.3525 |
| 6006 | 0 | 0 | 190.774 | 0 | 0 | 8.3565 | -3693.27 | 3.9772 |
| 6007 | 0 | 0 | 95.1 | 13.1 |  | 0 | -3757.29 | 3.6255 |
| 6008 | 0 | 0 | 407.0810665 | 0.640649844 |  | 0.1498 | -3722.92 | 3.3242 |
| 6009 | 328 | 0 | 0 | 0 | 77.318 | 0 | -3352.44 | 3.1553 |
| 6010 | 0 | 0 | 427.689 | 19.796 | 22.99525 | 0.255333333 | -2883.07 | 2.9387 |
| 6011 | 0 | 0 | 0 | 0 | 0.245 | 0.460666667 | -3371.97 | 2.787 |
| 6012 | 0 | 0 | 1.3 | 0 | 0.153333333 | 27.07 | -3390.47 | 2.5552 |
| 6013 | 0 | 438 | 37.057 | 3.227 | 1.176 | 0.0635 | -3186.41 | 2.1398 |
| 6014 | 0 | 0 | 1533 | 0 |  | 94.33275 | -3395.34 | 1.7578 |
| 6015 |  |  |  |  |  |  | -2949.98 | 1.4837 |
| 6016 | 0 | 0 | 0 | 0 |  | 1284.078667 | -2218.35 | 1.4878 |
| 6017 | 0 | 0 | 0 | 0 |  | 0 | -5988.93 | 1.7753 |
| 6018 |  |  |  |  |  |  | -4372.89 | 1.9612 |
| 6019 |  |  |  |  |  |  | -4613.28 | 1.9063 |
| 6184 |  |  |  |  |  |  | -4072.90 | 5.6987 |
| 6185 |  |  |  |  |  |  | -3913.55 | 5.2725 |
| 6186 |  |  |  |  | 0 | 0.028 | -3687.86 | 4.7643 |
| 6187 | 0 | 0 | 47.999 | 7 |  | 3.293 | -3796.92 | 4.259 |
| 6188 |  |  |  |  |  |  | -3473.75 | 3.8755 |
| 6189 | 0 | 0 | 6.261557895 | 0 | 0 | 0.149076923 | -3322.08 | 3.5843 |
| 6190 | 75.285 | 6.845 | 1.092 | 0 | 77.92942857 | 0 | -3174.15 | 3.2533 |
| 6191 | 547.5 | 0 | 250.398 | 0 | 0.740208333 | 12.0416 | -2737.82 | 3.061 |
| 6192 | 0 | 0 | 438 | 0 | 39.89092308 | 20.5298 | -1633.59 | 2.7897 |
| 6193 |  |  |  |  | 0.0174 | 26.467 | -3208.07 | 2.288 |
| 6194 | 0 | 0 | 0 | 0 | 0.3 | 622.3433333 | -3651.96 | 1.8875 |
| 6195 | 0 | 0 | 0 | 0 |  | 1949.9885 | -4022.88 | 1.764 |
| 6196 | 0 | 0 | 1.68 | 0 |  | 0 | -5669.97 | 1.979 |
| 6197 | 0 | 0 | 34.888 | 0 |  | 0 | -5279.56 | 2.3095 |
| 6198 |  |  |  |  |  |  | -3987.49 | 2.3257 |
| 6199 |  |  |  |  |  | 0.135 | -4796.38 | 2.2743 |
| 6365 |  |  |  |  |  |  | -4074.98 | 5.6805 |
| 6366 | 0 | 0 | 9.65 | 0 |  | 14.018 | -3960.92 | 5.1873 |
| 6367 | 0 | 0 | 0 | 0 |  | 1.2 | -3771.75 | 4.636 |
| 6368 | 0 | 0 | 73.4 | 0.266666667 |  | 2.727 | -3243.97 | 4.201 |
| 6369 | 164.256 | 177.945 | 202.574 | 5.476 | 14.921 | 0.072333333 | -2779.50 | 3.8567 |
| 6370 | 0 | 250.227619 | 19.93513043 | 0.904347826 | 0.226125 | 0.059 | -1210.66 | 3.4343 |
| 6371 | 0 | 0 | 15.63857143 | 0 | 13.0931 | 324.669 | -195.20 | 2.961 |
| 6372 | 0 | 0 | 7.266666667 | 0 | 48.0934386 | 210.3567167 | -1332.10 | 2.7443 |
| 6373 | 0 | 0 | 2.957 | 0 | 1121.352444 | 17.52585714 | -4454.36 | 2.401 |
| 6374 | 0 | 0 | 0 | 0 |  | 3.789 | -5408.35 | 2.0605 |
| 6375 | 0 | 0 | 0 | 0 |  | 16.841 | -5720.15 | 2.0487 |
| 6376 | 0 | 0 | 0 | 0 |  | 0 | -5577.17 | 2.3667 |
| 6377 |  |  |  |  |  |  | -4529.49 | 2.658 |
| 6378 |  |  |  |  |  |  | -4026.36 | 2.6573 |
| 6379 |  |  |  |  |  |  | -4443.69 | 2.6377 |
| 6380 |  |  |  |  |  | 1.609333333 |  |  |
| 6546 |  |  |  |  |  |  | -2883.23 | 5.5978 |
| 6547 |  |  |  |  |  |  | -2573.33 | 5.0183 |
| 6548 | 0 | 0 | 73 | 0 |  | 0.074666667 | -1689.71 | 4.514 |
| 6549 | 0 | 0 | 12.16 | 0 | 0.384 | 3.413875 | -1720.12 | 4.094 |
| 6550 | 13.03253968 | 54.69354839 | 198.3312698 | 0.868888889 | 34.14123529 | 12.82376101 | -2313.07 | 3.7217 |
| 6551 | 3.158846154 | 0.0124 | 15.86129032 | 0 | 769.8816296 | 15.80078481 | -1441.10 | 3.2978 |
| 6552 | 0 | 0 | 56.49133333 | 0.630444444 | 43.97328571 | 70.38011538 | -3096.06 | 3.0815 |
| 6553 | 0 | 0 | 13.74 | 6.75 | 497.4076667 | 0.8535 | -2934.88 | 2.7775 |
| 6554 | 0 | 0 | 8.045 | 0 |  | 0.0685 | -3757.40 | 2.5515 |
| 6555 | 0 | 0 | 0 | 0 |  | 0 | -4073.32 | 2.6458 |
| 6556 | 63.878 | 0 | 0 | 0 |  | 0 | -4806.90 | 3.0408 |
| 6557 | 0 | 0 | 0 | 0 |  | 0 | -4487.49 | 3.2455 |
| 6558 |  |  |  |  |  |  | -4028.75 | 3.2962 |
| 6559 | 0 | 0 | 0 | 1.2 |  | 0 | -4311.70 | 3.302 |
| 6727 |  |  |  |  |  |  | -3097.99 | 5.5847 |
| 6728 | 0 | 0 | 2 | 0 |  | 0.12 | -3041.87 | 5.113 |
| 6729 | 0 | 0 | 27.53879671 | 0 | 0 | 1.078705882 | -3657.93 | 4.5763 |
| 6730 | 0 | 0 | 487.7306758 | 0.224613333 | 0.0822 | 0 | -3737.19 | 4.2527 |
| 6731 | 0 | 0 | 31.40365404 | 0 | 19.42816667 | 25.97871429 | -2968.14 | 3.9365 |
| 6732 | 0 | 0 | 3 | 1 | 0 | 0.296333333 | -3812.49 | 3.8043 |
| 6733 | 0 | 0 | 0 | 0 |  | 0 | -2777.46 | 3.427 |
| 6734 | 0 | 0 | 0 | 0 |  |  | -3306.23 | 3.285 |
| 6735 | 0 | 0 | 0 | 0 |  | 1.342 | -3505.75 | 3.436 |
| 6736 | 0 | 0 | 4.003 | 0 |  | 75.1095 | -4454.61 | 3.837 |
| 6737 |  |  |  |  |  |  | -3562.83 | 3.965 |
| 6738 |  |  |  |  |  |  | -4461.64 | 3.9897 |
| 6739 |  |  |  |  |  |  | -4510.08 | 3.9593 |
| 6908 |  |  |  |  |  |  | -2072.31 | 5.806 |
| 6909 | 0 | 0 | 42.66666667 | 0 | 0 | 0 | -3289.35 | 5.173 |
| 6910 | 0 | 0 | 0 | 0 |  | 0.224 | -4034.65 | 4.849 |
| 6911 | 0 | 0 | 9.133333333 | 0 |  | 0 | -4424.62 | 4.6605 |
| 6912 |  |  |  |  |  |  | -4333.95 | 4.4853 |
| 6913 | 0 | 0 | 0 | 0 |  | 0.173 | -2809.96 | 4.0197 |
| 6914 |  |  |  |  |  |  | -3622.29 | 3.971 |
| 6915 | 0 | 0 | 0 | 0 |  | 0 | -4254.27 | 4.062 |
| 6916 |  |  |  |  |  |  | -3875.91 | 4.373 |
| 6917 |  |  |  |  |  | 0.116 | -3642.33 | 4.4795 |
| 6918 |  |  |  |  |  |  | -4508.17 | 4.4927 |
| 6919 |  |  |  |  |  |  | -4513.12 | 4.4313 |
| 7092 |  |  |  |  |  |  | -4201.84 | 5.767 |
| 7093 | 0 | 0 | 0 | 0 |  | 0 | -4734.32 | 5.4432 |
| 7094 |  |  |  |  |  |  | -4570.84 | 5.2765 |
| 7095 |  |  |  |  |  |  | -4657.17 | 5.1918 |
| 7096 |  |  |  |  |  |  | -3541.46 | 5.2632 |
| 7097 |  |  |  |  |  |  | -4274.64 | 5.3407 |
| 7098 |  |  |  |  |  |  | -4495.84 | 5.3335 |
| 7099 |  |  |  |  |  |  | -4315.31 | 5.1827 |
