# Supplementary material for: Habitat partitioning in Antarctic krill: Spawning hotspots and nursery areas
Source: PLoS One. 2019 Jul 24;14(7):e0219325. doi: 10.1371/journal.pone.0219325 (PMC6655634; doi:10.1371/journal.pone.0219325)
Supplement: S5 Table — This information was used in the construction of Fig 6. Blank cells contain no data. (DOCX) [file pone.0219325.s005.docx]

| grid_1x2_ID | egg_stations | naupmeta_stations | calyp_stations | fur_stations | ab_early_stations | ab_late_stations | lf_early_stations | lf_late_stations |
| --- | --- | --- | --- | --- | --- | --- | --- | --- |
| 2226 |  |  | 2 | 2 |  |  |  |  |
| 2227 |  |  | 1 | 1 |  |  |  |  |
| 2228 |  |  | 1 | 1 |  |  |  |  |
| 2229 |  |  | 2 | 2 |  | 2 |  | 1 |
| 2231 |  |  | 3 | 3 |  |  |  |  |
| 2232 |  |  | 2 | 2 |  |  |  |  |
| 2402 |  |  | 1 | 1 |  |  |  |  |
| 2403 |  |  | 2 | 2 |  |  |  |  |
| 2404 |  |  | 3 | 3 |  |  |  |  |
| 2405 |  |  | 10 | 10 |  |  |  |  |
| 2406 |  |  | 3 | 3 |  |  |  |  |
| 2413 |  |  | 1 | 1 |  |  |  |  |
| 2414 |  |  | 1 | 1 |  |  |  |  |
| 2415 |  |  |  |  | 1 |  | 2 |  |
| 2580 |  |  | 2 | 2 |  |  |  |  |
| 2581 |  |  | 1 | 1 |  |  |  |  |
| 2582 |  |  | 1 | 1 |  |  |  |  |
| 2585 |  |  | 1 | 1 |  |  |  |  |
| 2596 |  |  | 3 | 3 | 2 | 2 | 1 | 1 |
| 2759 |  |  | 1 | 1 |  |  |  |  |
| 2770 |  |  | 3 | 3 |  |  |  |  |
| 2771 |  |  | 3 | 3 |  |  |  |  |
| 2772 |  |  | 4 | 4 |  |  |  |  |
| 2773 |  |  | 2 | 2 |  |  |  |  |
| 2777 |  |  | 2 | 2 |  | 2 |  | 4 |
| 2950 |  |  | 1 | 1 |  |  |  |  |
| 2952 |  |  | 3 | 3 |  |  |  |  |
| 2958 |  |  | 1 | 1 | 1 |  | 1 |  |
| 2959 |  |  | 2 | 2 |  |  |  |  |
| 3113 |  |  |  |  |  | 2 |  | 1 |
| 3139 |  |  |  |  |  | 1 |  | 3 |
| 3140 |  |  |  |  |  |  | 1 |  |
| 3291 |  |  |  |  |  | 1 |  | 1 |
| 3833 |  |  |  |  |  | 10 |  | 1 |
| 3834 |  | 1 | 2 | 2 |  |  |  |  |
| 3835 |  | 1 | 11 | 12 |  | 39 |  | 2 |
| 3836 |  |  | 2 | 2 |  |  |  |  |
| 4012 |  | 1 | 1 | 1 |  |  |  |  |
| 4013 |  | 3 | 3 | 4 |  | 12 |  | 1 |
| 4014 |  | 4 | 5 | 8 | 99 | 99 | 2 | 2 |
| 4015 |  | 1 | 11 | 12 |  | 96 |  | 15 |
| 4192 |  | 2 | 2 | 2 |  | 1 |  | 1 |
| 4193 |  | 3 | 6 | 6 |  | 82 |  | 2 |
| 4194 |  | 3 | 13 | 15 |  | 85 |  | 10 |
| 4195 | 11 | 14 | 15 | 17 | 99 | 99 | 2 | 14 |
| 4196 |  |  | 3 | 4 |  | 89 |  | 10 |
| 4205 |  |  |  |  |  | 1 |  | 1 |
| 4207 |  |  | 1 | 1 |  |  |  |  |
| 4208 |  |  | 2 | 2 |  |  |  |  |
| 4373 |  | 2 | 2 | 2 |  | 1 |  | 1 |
| 4374 |  | 4 | 4 | 6 | 43 | 43 | 2 | 5 |
| 4375 |  | 4 | 4 | 6 | 117 | 117 | 2 | 5 |
| 4376 | 4 | 5 | 6 | 9 | 94 | 94 | 2 | 8 |
| 4377 |  | 1 | 1 | 1 |  |  |  |  |
| 4384 |  |  |  |  |  | 2 |  | 3 |
| 4385 |  |  |  |  |  | 9 |  | 2 |
| 4386 |  |  |  |  |  | 4 |  | 3 |
| 4387 |  |  | 1 | 1 |  |  |  |  |
| 4388 |  |  | 1 | 1 |  |  |  |  |
| 4554 |  | 3 | 3 | 3 |  | 1 |  | 1 |
| 4555 |  | 2 | 2 | 4 | 53 | 53 | 2 | 7 |
| 4556 | 1 | 5 | 5 | 6 | 85 | 85 | 1 | 5 |
| 4557 | 10 | 12 | 13 | 16 | 199 | 199 | 4 | 19 |
| 4558 | 2 | 5 | 5 | 8 | 49 | 49 | 2 | 13 |
| 4562 |  |  |  |  |  | 7 |  | 10 |
| 4563 |  |  |  |  |  | 2 |  | 2 |
| 4564 |  |  |  |  |  | 3 |  | 3 |
| 4566 |  |  | 1 | 1 |  |  |  |  |
| 4567 |  |  | 1 | 1 |  | 1 |  | 1 |
| 4568 |  |  | 1 | 1 |  | 2 |  | 2 |
| 4735 |  | 3 | 3 | 3 | 3 | 3 | 1 | 1 |
| 4736 | 2 | 5 | 6 | 8 | 56 | 56 | 2 | 6 |
| 4737 | 3 | 6 | 7 | 8 | 24 | 24 | 3 | 10 |
| 4738 | 1 | 6 | 9 | 16 | 39 | 39 | 9 | 24 |
| 4739 | 11 | 13 | 26 | 33 | 128 | 128 | 24 | 37 |
| 4740 | 4 | 6 | 12 | 13 | 66 | 66 | 16 | 13 |
| 4741 | 7 | 7 | 10 | 10 | 14 | 14 | 5 | 8 |
| 4742 |  |  | 3 | 4 | 17 | 17 | 2 | 16 |
| 4743 | 2 | 1 | 2 | 2 |  | 4 |  | 3 |
| 4744 |  |  | 1 | 1 |  | 1 |  | 2 |
| 4745 | 6 | 6 | 10 | 10 |  | 3 |  | 4 |
| 4746 |  |  | 2 | 2 |  |  |  |  |
| 4747 |  |  | 1 | 1 |  |  |  |  |
| 4748 |  |  | 1 | 1 |  |  |  |  |
| 4749 |  |  | 1 | 1 |  |  |  |  |
| 4750 |  |  | 1 | 1 |  |  |  |  |
| 4752 |  |  | 1 | 1 |  |  |  |  |
| 4753 |  |  | 1 | 1 |  |  |  |  |
| 4754 |  |  | 1 | 1 |  |  |  |  |
| 4755 |  |  | 1 | 1 |  |  |  |  |
| 4759 |  |  | 11 | 11 |  |  |  |  |
| 4916 | 1 | 2 | 3 | 4 |  | 7 |  | 2 |
| 4917 | 2 | 3 | 7 | 7 | 7 | 7 | 2 | 2 |
| 4918 | 1 | 7 | 11 | 14 | 68 | 68 | 4 | 14 |
| 4919 | 5 | 5 | 27 | 31 | 168 | 168 | 13 | 36 |
| 4920 | 6 | 5 | 22 | 24 | 219 | 219 | 21 | 36 |
| 4921 | 9 | 12 | 38 | 42 | 307 | 307 | 41 | 52 |
| 4922 | 2 | 3 | 12 | 15 | 102 | 102 | 30 | 36 |
| 4923 | 1 | 1 | 9 | 9 | 20 | 20 | 2 | 19 |
| 4924 | 3 | 1 | 9 | 9 |  | 5 |  | 14 |
| 4925 | 4 | 3 | 6 | 6 | 5 | 5 | 2 | 6 |
| 4926 |  |  |  |  | 5 |  | 1 |  |
| 4927 | 1 |  | 1 | 1 |  | 3 |  | 1 |
| 4928 |  |  | 1 | 1 |  |  |  |  |
| 4930 |  |  |  |  |  |  | 1 |  |
| 4931 |  |  |  |  | 1 |  | 1 |  |
| 4935 | 1 | 1 | 1 | 1 |  |  |  |  |
| 5095 | 2 | 1 | 2 | 2 |  |  |  |  |
| 5096 | 1 |  | 1 | 1 |  |  |  |  |
| 5097 |  | 1 | 2 | 2 |  |  |  |  |
| 5098 | 5 | 6 | 7 | 9 | 40 | 40 | 1 | 2 |
| 5099 | 1 | 5 | 11 | 12 | 157 | 157 | 2 | 8 |
| 5100 | 2 | 2 | 15 | 16 | 215 | 215 | 9 | 15 |
| 5101 | 4 | 6 | 26 | 41 | 475 | 475 | 86 | 67 |
| 5102 | 17 | 18 | 42 | 63 | 562 | 562 | 86 | 95 |
| 5103 | 3 | 2 | 15 | 15 | 174 | 174 | 20 | 14 |
| 5104 | 1 |  | 7 | 7 |  | 6 |  | 6 |
| 5105 | 3 | 2 | 5 | 5 | 9 | 9 | 1 | 5 |
| 5106 | 1 | 1 | 3 | 3 | 11 | 11 | 2 | 3 |
| 5107 | 1 | 1 | 2 | 2 |  | 15 |  | 1 |
| 5108 | 1 |  | 3 | 3 |  | 5 |  | 2 |
| 5110 |  |  | 1 | 1 |  |  | 2 |  |
| 5111 | 2 | 1 | 2 | 2 |  | 10 | 6 | 1 |
| 5112 |  |  | 1 | 1 |  |  |  |  |
| 5114 | 1 | 1 | 1 | 1 |  | 2 |  | 1 |
| 5277 | 1 |  | 1 | 2 |  | 3 |  | 3 |
| 5278 |  |  | 1 | 1 |  |  | 1 |  |
| 5279 | 1 |  | 2 | 2 |  | 29 |  | 2 |
| 5280 |  | 1 | 5 | 7 | 71 | 71 | 10 | 5 |
| 5281 | 2 | 6 | 16 | 23 | 449 | 449 | 48 | 26 |
| 5282 | 19 | 25 | 49 | 70 | 637 | 637 | 107 | 145 |
| 5283 | 1 | 1 | 5 | 5 | 220 | 220 | 22 | 18 |
| 5284 | 6 | 6 | 8 | 8 | 4 | 4 | 2 | 6 |
| 5285 | 4 | 21 | 25 | 25 | 32 | 32 | 3 | 6 |
| 5286 | 3 | 2 | 6 | 6 | 47 | 47 | 8 | 10 |
| 5287 | 1 | 14 | 20 | 20 |  | 58 | 4 | 3 |
| 5288 | 5 | 4 | 7 | 7 | 19 | 19 | 3 | 7 |
| 5289 | 2 | 1 | 2 | 2 | 13 | 13 | 5 | 2 |
| 5290 | 1 |  | 1 | 1 | 13 |  | 10 |  |
| 5291 |  |  |  |  |  |  | 1 |  |
| 5292 | 1 |  | 1 | 1 |  | 5 |  | 2 |
| 5293 | 1 | 1 | 1 | 1 |  |  |  |  |
| 5294 | 1 |  | 1 | 1 |  |  |  |  |
| 5295 | 2 | 1 | 2 | 2 |  |  |  |  |
| 5296 | 1 |  | 1 | 1 |  | 1 |  | 1 |
| 5297 | 1 |  | 1 | 1 |  | 1 |  | 1 |
| 5460 |  |  | 1 | 1 |  |  |  |  |
| 5461 | 2 | 1 | 2 | 2 | 75 | 75 | 4 | 1 |
| 5462 | 1 | 1 | 2 | 2 | 97 | 97 | 4 | 3 |
| 5463 |  |  | 6 | 6 | 50 | 50 | 2 | 5 |
| 5464 | 3 | 3 | 9 | 9 | 15 | 15 | 1 | 5 |
| 5465 | 1 | 1 | 4 | 4 | 71 | 71 | 5 | 24 |
| 5466 | 1 | 1 | 6 | 6 | 22 | 22 | 3 | 11 |
| 5467 | 1 | 14 | 21 | 21 |  | 39 | 3 | 10 |
| 5468 | 1 |  | 4 | 4 |  | 9 | 1 | 54 |
| 5469 | 1 | 1 | 1 | 1 |  |  | 2 |  |
| 5470 | 1 |  | 1 | 1 | 13 | 13 | 9 | 4 |
| 5471 | 2 | 1 | 2 | 2 | 8 | 8 | 3 | 3 |
| 5472 | 1 |  | 1 | 1 |  | 3 |  | 4 |
| 5473 | 2 | 1 | 2 | 2 |  | 2 |  | 2 |
| 5475 |  |  | 1 | 1 |  |  |  |  |
| 5476 | 1 | 1 | 2 | 2 |  |  |  |  |
| 5477 | 2 |  | 2 | 2 |  | 2 |  | 3 |
| 5478 | 1 |  | 1 | 1 |  | 1 |  | 1 |
| 5479 |  |  | 1 | 1 |  |  |  |  |
| 5642 | 1 | 1 | 1 | 1 |  |  |  |  |
| 5644 | 1 | 1 | 7 | 7 |  | 7 |  | 5 |
| 5645 | 3 | 2 | 7 | 7 | 14 | 14 | 2 | 3 |
| 5646 | 2 | 1 | 3 | 3 | 9 | 9 | 3 | 2 |
| 5647 | 2 | 1 | 9 | 9 |  | 10 | 2 | 2 |
| 5648 | 1 | 15 | 22 | 22 |  | 13 |  | 7 |
| 5649 | 2 | 1 | 2 | 2 |  | 3 | 1 | 3 |
| 5650 |  |  |  |  |  |  | 3 |  |
| 5651 | 1 | 1 | 1 | 1 | 3 | 3 | 2 | 1 |
| 5652 | 2 | 2 | 3 | 3 |  |  |  |  |
| 5653 | 1 |  | 1 | 1 |  | 1 |  | 2 |
| 5655 | 3 | 2 | 3 | 3 |  | 2 |  | 4 |
| 5656 | 4 | 1 | 5 | 5 |  | 5 |  | 14 |
| 5657 | 1 |  | 1 | 1 |  | 1 |  | 6 |
| 5822 |  |  | 1 | 1 |  |  |  |  |
| 5824 | 1 | 3 | 5 | 5 |  | 14 |  | 1 |
| 5825 | 1 | 1 | 5 | 5 |  |  |  |  |
| 5826 |  |  |  |  |  |  | 1 |  |
| 5827 | 3 | 2 | 5 | 5 |  | 4 |  | 2 |
| 5828 | 2 | 1 | 4 | 4 |  | 10 |  | 3 |
| 5829 | 3 | 1 | 3 | 3 |  | 3 |  | 1 |
| 5830 |  |  | 1 | 1 |  |  |  |  |
| 5831 | 3 | 2 | 3 | 3 |  | 5 |  | 2 |
| 5832 | 1 |  | 1 | 1 |  | 4 |  | 2 |
| 5834 | 1 | 1 | 1 | 1 |  | 4 | 1 | 2 |
| 5835 | 2 | 1 | 2 | 2 |  | 1 |  | 2 |
| 5836 | 1 |  | 2 | 2 |  | 2 |  | 1 |
| 5838 | 1 |  | 1 | 1 |  | 2 |  | 1 |
| 5839 |  |  | 1 | 1 |  |  |  |  |
| 6004 | 1 | 1 | 1 | 1 |  |  |  |  |
| 6006 | 1 |  | 1 | 1 |  |  |  |  |
| 6007 | 1 | 1 | 2 | 2 |  |  |  |  |
| 6008 | 1 | 6 | 8 | 8 |  | 5 |  | 1 |
| 6009 | 1 | 1 | 1 | 1 |  | 2 |  | 1 |
| 6010 | 1 |  | 2 | 2 |  | 7 |  | 2 |
| 6011 | 1 | 1 | 3 | 3 |  |  |  |  |
| 6012 | 1 |  | 2 | 2 | 20 |  | 2 |  |
| 6013 | 2 | 1 | 2 | 2 |  | 3 |  | 4 |
| 6014 | 1 | 1 | 1 | 1 |  |  |  |  |
| 6016 | 2 |  | 4 | 4 |  | 3 |  | 2 |
| 6017 | 2 |  | 2 | 2 |  | 2 |  | 3 |
| 6187 | 1 |  | 2 | 2 |  |  |  |  |
| 6189 | 2 | 15 | 19 | 19 |  |  |  |  |
| 6190 | 4 | 4 | 5 | 5 |  |  | 3 |  |
| 6191 | 2 | 1 | 2 | 2 | 41 |  | 3 |  |
| 6192 | 1 | 1 | 1 | 1 | 18 |  | 1 |  |
| 6193 |  |  |  |  | 7 |  | 1 |  |
| 6194 | 2 | 1 | 2 | 2 |  | 4 | 1 | 1 |
| 6195 | 1 |  | 2 | 2 |  | 2 |  | 1 |
| 6196 | 3 |  | 3 | 3 |  | 3 |  | 5 |
| 6197 | 1 |  | 1 | 1 |  | 1 |  | 1 |
| 6366 | 1 |  | 1 | 1 |  |  |  |  |
| 6367 | 1 |  | 1 | 1 |  |  |  |  |
| 6368 | 2 | 1 | 3 | 3 |  | 2 |  | 1 |
| 6369 | 5 | 4 | 5 | 5 |  | 14 | 1 | 2 |
| 6370 | 22 | 21 | 23 | 23 | 34 | 34 | 1 | 45 |
| 6371 | 7 | 6 | 7 | 7 | 32 | 32 | 11 | 5 |
| 6372 | 14 | 12 | 15 | 15 | 117 | 117 | 16 | 36 |
| 6373 | 1 |  | 2 | 2 | 22 | 22 | 3 | 5 |
| 6374 | 2 | 1 | 3 | 3 |  | 2 | 1 | 1 |
| 6375 | 1 |  | 2 | 2 |  | 2 |  | 1 |
| 6376 | 1 |  | 1 | 1 |  |  |  |  |
| 6548 | 3 | 2 | 3 | 3 |  |  |  |  |
| 6549 | 9 | 9 | 9 | 9 |  | 18 | 1 | 4 |
| 6550 | 63 | 62 | 63 | 63 | 195 | 195 | 32 | 66 |
| 6551 | 26 | 25 | 31 | 31 | 106 | 106 | 30 | 97 |
| 6552 | 8 | 6 | 9 | 9 | 52 | 52 | 8 | 5 |
| 6553 | 5 | 5 | 5 | 4 | 20 |  | 2 |  |
| 6554 | 2 | 1 | 2 | 2 |  | 2 |  | 1 |
| 6555 | 1 |  | 1 | 1 |  |  |  |  |
| 6556 | 1 |  | 1 | 1 |  | 1 |  | 1 |
| 6557 | 1 |  | 1 | 1 |  |  |  |  |
| 6559 |  |  | 1 | 1 |  |  |  |  |
| 6728 | 2 | 1 | 3 | 3 |  |  |  |  |
| 6729 | 2 | 17 | 21 | 21 |  | 25 |  | 3 |
| 6730 | 2 | 10 | 12 | 12 |  | 24 |  | 4 |
| 6731 | 10 | 15 | 17 | 17 | 32 | 32 | 3 | 1 |
| 6732 | 1 |  | 2 | 2 |  |  |  |  |
| 6733 | 1 | 1 | 1 | 1 |  | 1 |  | 2 |
| 6734 | 1 | 1 | 1 | 1 |  |  |  |  |
| 6735 |  |  | 1 | 1 |  |  |  |  |
| 6736 | 1 |  | 2 | 2 |  | 2 |  | 1 |
| 6909 | 3 | 3 | 3 | 3 |  | 11 |  | 2 |
| 6910 | 1 |  | 1 | 1 |  | 1 |  | 2 |
| 6911 | 3 | 3 | 3 | 3 |  |  |  |  |
| 6913 | 1 |  | 1 | 1 |  | 1 |  | 2 |
| 6915 | 2 |  | 2 | 2 |  | 2 |  | 1 |
| 7093 |  | 8 | 9 | 9 |  |  |  |  |
